# Multi-dimensional interpretable deep learning-radiomics based on intra-tumoral and spatial habitat for preoperative prediction of thymic epithelial tumours risk categorisation

**DOI:** 10.2340/1651-226X.2025.42982

**Published:** 2025-03-13

**Authors:** Yuhua Yang, Jia Cheng, Can Cui, Huijie Huang, Meiling Cheng, Jiayi Wang, Minjing Zuo

**Affiliations:** a Department of Radiology, The Second Affiliated Hospital, Jiangxi Medical College, Nanchang University, Nanchang, China; b Jiangxi Provincial Key Laboratory of Intelligent Medical Imaging, Nanchang, China; c Department of Radiology, The First Affiliated Hospital of Gannan Medical University, Ganzhou, China

**Keywords:** Radiomics, deep learning, machine learning, thymoma

## Abstract

**Background and purpose:**

This study aims to develop and compare combined models based on enhanced CT-based radiomics, multi-dimensional deep learning, clinical-conventional imaging and spatial habitat analysis to achieve accurate prediction of thymoma risk classification.

**Materials and Methods:**

205 consecutive patients with thymoma confirmed by surgical pathology were recruited from three medical centers. Venous phase enhanced CT images were used to delineate the tumor, and radiomics, 2D and 3D deep learning models based on the whole tumor were established and feature extraction was performed. The tumors were divided into different sub-regions by K-means clustering method and the corresponding features were obtained. The clinical-conventional imaging data of the patients were collected and evaluated, and the univariate and multivariate analysis were used for screening. The above types of features were fused with each other to construct a variety of combined models. Quantitative indicators such as area under the receiver operating characteristic (ROC) curve (AUC) were calculated to evaluate the performance of the model.

**Results:**

The AUC of RDLCSM developed based on LightGBM classifier was 0.953 in the training cohort, 0.930 in the internal validation cohort, 0.924 and 0.903 in the two external validation cohorts, respectively. RDLCSM performs better than RDLM (AUC range: 0.831-0.890) and 2DLCSM (AUC range: 0.785-0.916) based on KNN. In addition, RDLCSM had the highest accuracy (0.818-0.882) and specificity (0.926-1.000).

**Interpretation:**

The RDLCSM, which combines whole-tumor radiomics, 2D and 3D deep learning, clinical-visual radiology, and subregional omics, can be used as a non-invasive tool to predict thymoma risk classification.

## Introduction

With the widespread use of computed tomography (CT) during chest examination, incidental findings of anterior mediastinal mass have become more common. The Framingham Heart Study revealed anterior mediastinal masses in 0.9% of 2,571 patients undergoing chest CT scans [1]. Thymoma is the most common tumour in anterior mediastinal mass [2]. The most common age of patients is between 35 and 70 years, with no statistically significant incidence differences between genders. About one third of patients have myasthenia gravis [3]. For early-stage localised thymoma, the 5-year survival rate of patients is about 94%, while for those with accumulated adjacent organ or distant metastasis, the 5-year survival rate drops to about 56% and 78%, respectively [4]. Based on the consensus of the International Thymic Malignancy Interest Group regarding thymic tumours, all types of thymomas are considered to have malignant potential except for the nodular type thymomas and microthymomas with lymphoid stroma [5]. The World Health Organization (WHO) classifies thymic epithelial tumours (TET) into types A, AB, B1, B2, and B3 thymomas and thymic carcinoma (TC) based on the morphology of epithelial cells and the ratio of lymphocytes to epithelial cells. Jeong et al. simplified the classification of thymomas based on tumour behaviour and prognosis into low-risk thymomas (LRT) (types A, AB, and B1) and high-risk thymomas (HRT) (types B2 and B3) [6]. Histological subtypes are important in guiding treatment options. For patients with LRT, the treatment is usually complete surgical resection without adjuvant or neoadjuvant chemotherapy. In contrast, HRT is often difficult to remove completely due to its more aggressive nature. Comprehensive treatment, including chemotherapy, radiotherapy, and/or surgical resection is required [7]. Thymoma histological classification, completeness of tumour resection, Masaoka-Koga staging and tumour node metastasis (TNM) staging system have been recognised as independent prognostic factors [8]. Tumour recurrence and mortality rates of type B2 and B3 thymoma patients are higher than those of other types [9]. Therefore, accurate preoperative classification is helpful to formulate individualised treatment methods and improve the prognosis of patients with thymoma.

Needle biopsy is a reliable method for the diagnosis of thymoma; however, smaller biopsy specimens may not represent the whole tumour, and deep biopsy is an invasive method with a risk of complications and transpleural puncture may lead to tumour implantation [10]. Medical imaging plays an important role in the preoperative diagnosis and follow-up monitoring of thymoma. Previous studies have shown that although traditional CT and magnetic resonance imaging (MRI) can provide detailed morphological information about tumour size, shape, homogeneity, and other characteristics; they have limited value in predicting tissue subtypes and they often cannot reliably detect invasion of adjacent structures at an early stage [11]. Qualitative imaging features such as tumour morphology or borders have considerable overlap among different subtypes of thymoma, making visual classification very difficult and subjective [12].

Radiomics extracts and analyses a large number of high-throughput features that are not visible to the naked eye from medical images, which can objectively quantify lesion heterogeneity and evaluate tumour detection, classification, and treatment response; thereby, overcoming the limitations of subjective visual image interpretation and providing effective support for medical decision-making. Radiomics has become an emerging field of precision medicine [13]. Recently, deep learning has achieved excellent performance in various medical image analysis tasks. It can directly and automatically learn task-related features from the input medical images, eliminating the requirement for accurate lesion boundaries of the input images [14]. Deep learning features have high stability and thus perform well in medical tasks across multiple medical centres. For medical deep learning applications with small samples, transfer learning is often used to overcome the limitation of data resources [15]. Several studies have developed fusion models integrating radiomics and deep learning, showing promising prospects for multidisciplinary fusion methods [16]; however, most rely on single 2D or 3D deep learning technologies, and the impact of multidimensional deep learning-radiomics combinations on model performance has not yet been clearly explored. Previous radiomics and deep learning methods assumed that the heterogeneity of the whole tumour was uniformly distributed, ignoring the local phenotypic differences within the tumour. Unlike previous methods, a new method called habitat imaging can explore the heterogeneity of different regions within a tumour by dividing the entire tumour into subregions containing similar voxels. Habitat imaging analysis has been proven to have excellent predictive ability for the treatment efficacy of oesophageal and breast cancer [17].

Currently, most studies on thymoma risk classification only use radiomics, and often ignore clinical-visual radiological features and the value of deep learning in them. A few studies using deep learning only used a single convolutional neural network based on 2D or 3D dimensions [18], and most of the studies were conducted in a single centre with a small sample size (<200).

To overcome these limitations, this study included thymoma patients (>200) from three different medical centres. Multi-dimensional deep learning, radiomics, clinical-visual radiological, and habitat imaging features were fused. Therefore, the risk assessment of thymoma can be performed more comprehensively and accurately. Although radiologists who perform manual segmentation are experienced, it is still impossible to avoid time-consuming processes and issues such as significant inter-observer and intra-observer variability. To mitigate these problems, deep learning has achieved great success in the automatic segmentation of medical images in recent years. Owing to the development of machine learning, we trained an automatic thymoma segmentation model based on chest enhanced CT.

## Materials and methods

### Patient selection

From October 2018 to August 2024, 377 patients with suspected thymic mass by CT examination in three medical centres were retrospectively enrolled in this study. The WHO histological classification of thymoma was determined according to the operation and pathological results.

Inclusion criteria were as follows: (1) lesion diameter > 5 mm; (2) chest enhanced CT scan was performed within 30 days before surgery; and (3) no treatment was taken before CT scan. Exclusion criteria were as follows: (1) poor image quality due to artifacts or other reasons; (2) no thin-slice CT scan was performed; and (3) the patients lacked complete imaging and clinical data.

**Figure 1:**
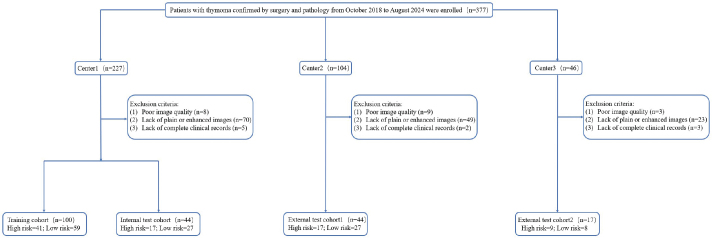
Flow diagram of cohort selection.

A total of 205 patients were finally included, among which the thymoma patients from the Second Affiliated Hospital of Nanchang University with the largest data volume (*n* = 144) were divided into training group and partial validation group according to the ratio of 7:3. Two smaller datasets from the First Affiliated Hospital of Gannan Medical University (*n* = 44) and the Affiliated Hospital of Jiujiang University (*n* = 17) constituted an independent external validation cohort. Figure 1 summarises the patient enrolment process in detail.

To verify whether the number of patients included in this study met the minimum sample size required to detect significant differences between different risk levels of thymoma, we used the G*Power software developed by Dusseldorf University (www.gpower.hhu.de, version 3.1.9.7.143) for power analysis. The statistical test was set to an independent samples *t*-test, and the type of power analysis was set to *a priori* compute the required sample size. The allocation ratio was 1.44 (LRT sample size *n* = 121 / HRT sample size *n* = 84). The effect size, α error probability, and power (1 – β error probability) were set to 0.5, 0.05, and 0.8, respectively. The output results indicated that the minimum sample sizes required for sample groups 1 and 2 were 54 and 78, respectively, with a minimum total sample size of 132. Therefore, the sample size for this study meets the requirements for thymoma risk level differentiation.

### Image acquisition and tumour segmentation

A chest CT scan was performed with the patient in the supine position with a deep breath hold after full inspiration. The scanning range was from the level of the thoracic entrance to the level of the adrenal gland. A conventional plain CT scan was first performed, and 1.3–1.5 mL/Kg of non-ionic iodinated contrast material was injected into the patient’s antecubital vein at a rate of 2.5–3.0 mL/s. Delayed imaging times were 30–40 seconds and 60–70 seconds in arterial and venous phases, respectively. The tube voltage was 120 kV and the tube current was 150 mA, 200 mA or adaptive; the other detailed parameters are shown in Supplementary Table 1.

Each patient image obtained from picture archiving and communication system (PACS) was imported into 3D Slicer software (version 5.2.2). In order to clearly show the structure of the anterior mediastinum, the CT image window width was set to 400 HU, and the window level was set to 40 HU. Tumour boundaries were delineated to create regions of interest (ROI) by two radiologists with extensive experience in chest imaging who were unaware of histopathological findings. Previous studies have shown that anterior mediastinal tumours will show relatively clear boundaries in venous phase images [19], and venous phase images are widely used for radiomics analysis of soft tissue tumours (such as gastric cancer, renal tumour and hepatocellular carcinoma) [20]. Therefore, thin-layer images in venous phase were selected for lesion segmentation in this study.

### Radiologist assessment

Baseline clinical characteristics were obtained from our medical records and included the patient’s age, sex, presence or absence of muscle weakness, presence or absence of other chest diseases, and chest symptoms (chest pain, cough, dyspnoea, etc.). Clinical-visual radiological features were assessed by two radiologists with 10 years of experience, who were blinded to the histologic classification and model diagnosis of the patients: (1) Primary site (middle or side); (2) Lobulated (absent or present); (3) Density uniformity (uniform or nonuniform); (4) Calcification (absent or present); (5) Shape (regular or irregular); (6) Boundary clarity (clear or vague); (7) 2D maximum axis diameter (The maximum long axis of the maximum cross section at the axial position); (8) 3D maximum diameter (The longest axis of the lesion in three dimensions); (9) Pre-contrast CT attenuation in HU; (10) Post-contrast CT attenuation in HU; and (11) Change of CT value in HU. Examples of evaluation are shown in Supplementary Figure 1.

### Spatial tumour habitats

In order to further identify the intra-tumour heterogeneity, we used the K-means algorithm to cluster the venous phase lesion images of all patients separately. The Euclidean distance between each voxel was used as the metric to minimise the sum of the distance between each object and the centre of its cluster. The voxels contained in each cluster had similar HU value intensity and texture information. Since the optimal number of clusters in the dataset is not a fixed value, this study set the number of clusters to range from 2 to 5, and evaluated the clustering results by calculating three metrics: Silhouette Coefficient, Calinski-Harabasz Index, and Davies-Bouldin Index, from which the optimal number of clusters was selected. Ultimately, each tumour was divided into spatially distinct subregions.

### Auto-segmentation

In this work, we trained an automatic segmentation framework for thymomas based on the DeepLabv3 deep neural network. The 144 patient images from centre 1 were divided into a training set and a validation set (the parameters of the model were adjusted by the validation set) at a ratio of 3:1. Patients from two external validation cohorts (*n* = 61) were used as test sets to evaluate model performance. All original images and ROIs were cropped to 256 × 256, with a batch size set to 8. The segmentation performance on the test set was assessed using Dice score, intersection-over-union (IoU), precision, and recall.

### Radiomics feature extraction

In image processing, variations in scanning devices and parameters can result in images with different resolutions and pixel sizes, and we used resampling to a 1 × 1 × 1 mm^3^ voxel size to ensure that all images were compared and analysed at uniform resolution. We used the ‘Pyradiomics’ package in Python 3.9.12 to analysis each tumour as a whole and in its subregion [21]. The LoG (Laplacian of Gaussian) sigma was set to 1.0, 2.0, and 3.0, the bin width was set to 25, and two different filters were applied: wavelet and LoG, for feature extraction from both the original and transformed whole tumour images.

Finally, a total of 1,106 radiomics features were extracted from each tumour: (1) 14 shape features; (2) 18 first order features; (3) 22 Gray Level Co-occurrence Matrix (GLCM) features; (4) 14 Gray Level Dependence Matrix (GLDM) features; (5) 16 Gray Level Run Length Matrix (GLRLM) features; (6) 16 Gray Level Size Zone Matrix (GLSZM) features; (7) 5 adjacent Gray Tone Difference Matrix (NGTDM) features; (8) 273 LoG filtering features; and (9) 728 wavelet filtering features. A total of 107 radiomics features were extracted from each habitat: (1) 14 shape features; (2) 18 first order features; (3) 24 GLCM features; (4) 14 GLDM features; (5) 16 GLRLM features; (6) 16 GLSZM features; and (7) 5 Neighbourhood gray-tone difference matrix (NGTDM) features.

### Radiomics feature selection and model construction

Radiomic features were standardised using *Z*-score normalisation, which transforms the measured values of the radiomic features to have a mean of 0 and a variance of 1 [22]. A random selection of 30 patients’ venous-phase CT images was re-segmented to calculate the Intra-observer Correlation Coefficient (ICC) to identify features with high stability, where features with an ICC greater than 0.75 were considered to have good consistency and they were retained [23]. We further processed features with strong correlations (removing one of the two features with a Pearson correlation coefficient > 0.9) [24]. The Least Absolute Shrinkage and Selection Operator (LASSO) was used to construct a penalty function λ to shrink some regression coefficients, forcing them to be zero, thus retaining stable features, and 10-fold cross-validation was employed to determine the optimal λ value based on the minimum criterion.

We used K-Nearest Neighbors (kNN), Light Gradient Boosting Machine (LightGBM), Multilayer Perceptron (MLP), and three machine learning classifiers to construct the radiomics model (RM). The process of feature selection and model establishment in this study was based on OneKey platform (http://www.medai.icu).

### Clinical-visual radiological feature selection

A univariate analysis was conducted on the 16 clinical-visual radiological features: patient age, gender, myasthenia, thoracic diseases, thoracic symptoms, primary site, lobulated, density uniformity, calcification, shape, boundary clarity, 2D maximum axis diameter, 3D maximum diameter, pre-contrast CT attenuation in HU, post-contrast CT attenuation in HU, and change of CT value in HU. Features with a *p* value < 0.05 were retained for multivariate logistic regression (LR) analysis.

### 2D and 3D deep learning model development, and feature extraction

For the 2D deep learning model (2DLM), with the window width set to 350 HU and the window level set to 40 HU, the largest ROI slice containing the whole lesion was selected on the axial view using a 2D rectangular border, and the input image was trimmed to 256 × 256. Four convolutional neural networks, Densenet161, GoogLeNet, Mobilenet_v2, and Resnet152, which had been pre-trained on the ImageNet dataset, were used for transfer learning. The Batch size was set to 128, the L2 regularisation strategy was used to prevent overfitting, Stochastic gradient descent (SGD) was used as the optimiser, and the initial learning rate was 0.01. The pre-processed images were input for training to construct a 2DLM.

We recognise the limitations of 2D deep learning in obtaining 3D lesion information, Therefore, four 3D deep learning networks Densenet201, Resnet18, ShuffleNet, and Vision Transformer (ViT) were used for migration based on Onekey platform (http://www.medai.icu). In order to meet the requirements of the input images, all images were trimmed to 64 × 64 × 48, the batch size was set to 4, Adam was used as the optimiser, the initial learning rate was 0.001, and 100 times (epoch = 100) training was performed to construct a 3D deep learning model (3DLM).

Since our goal was to extract features, we removed the last fully connected layer of the pre-trained network, used the penultimate average pooling layer (Avgpool) for feature extraction. A total of 2,048 deep learning features were extracted for each 2D and 3DLM, and we used principal component analysis (PCA) to reduce the feature dimension to 32. Finally, the pair of 2D and 3D deep learning features were screened out by LASSO algorithm for subsequent model fusion. All GPUs used for deep learning network training are NVIDIA RTX 3060Ti.

### Model fusion

In addition to the individual models constructed above (RM, 2DLM, 3DLM), this study aimed to further explore the value of radiomics, clinical-visual radiological, multi-dimensional deep learning, and tumour subregions in the risk classification of thymomas by building multiple combined models through prefusion of different features.

There are two types of feature fusion: R2DLM (radiomics + 2D deep learning), R3DLM (radiomics + 3D deep learning), DLM (2D deep learning + 3D deep learning), and CSM (clinical-visual radiological + tumour subregion). For three types of feature fusion, we have RDLM (radiomics + 2D deep learning + 3D deep learning), RCSM (radiomics + clinical-visual radiological + tumour subregion), 2DLCSM (2D deep learning + clinical-visual radiological + tumour subregion), and 3DLCSM (3D deep learning + clinical-visual radiological + tumour subregion). For five types of feature fusion, we have RDLCSM (radiomics + 2D deep learning + 3D deep learning + clinical-visual radiological + tumour subregion).

All of the above fusion models were evaluated by different machine learning classifiers such as LR, Support vector Machine (SVM), kNN, and other machine learning methods. LightGBM, Adaptive boosting (AdaBoost), MLP, Extra trees (ET) and others built models were used and their performance were compared, and the best classifier of each fusion model as a representative was selected.

### Subregion clustering

**Figure 2:**
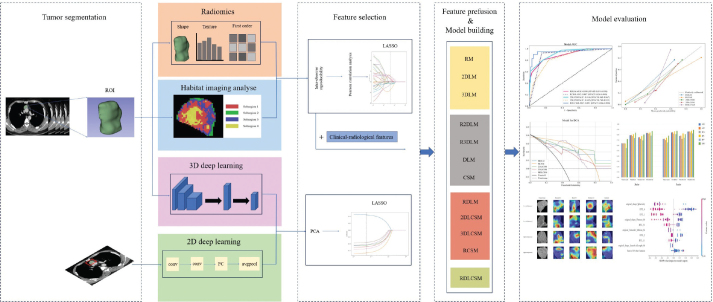
The workflow and global analysis pipeline in this paper. The radiologist delineated the region of interest (ROI) of the tumour, extracted the radiomics features, divided the habitat sub-regions, and trained the 3D deep learning. 2D deep learning model were constructed based on the maximum cross section of the lesion. The features were screened and fused to construct a variety of combination models. Finally, the models were evaluated, compared and visually interpreted.

The number of habitats was tested from 2 to 5, The most appropriate K value was selected by calculating Silhouette Coefficient, Calinski-Harabasz Index, and Davies-Bouldin Index (Supplementary Table 2). The Silhouette Coefficient is used to measure the relationship between the similarity within a sample and its own cluster and the dissimilarity of other clusters. The closer the value range is to 1, the more reasonable the clustering is. The Calinski-Harabasz index assesses the closeness of a cluster by calculating the ratio of the covariance matrix within and between clusters. A larger index indicates a larger difference between clusters and a higher similarity within clusters. The Davies-Bouldin index is used to measure the average similarity between each cluster and its most similar cluster. A smaller index indicates a more reasonable clustering. The results showed that when the cluster number is *K* = 4, it had the highest Calinski-Harabasz Index (561580.096) and the lowest Davies-Bouldin Index (0.8). Therefore, we divided the region of interest into four sub-regions. Figure 2 shows the habitat image with four colours representing different cluster subregions.

### Radiomics and subregion feature selection and modelling

Among the 1,106 radiomic features extracted from the entire tumour, 9 key features were ultimately identified following LASSO feature selection. The LASSO feature selection process and the variation curve of the penalty coefficient λ are shown in Supplementary Figure 3A and 3B. When the optimal λ is 0.045, the model includes the fewest features while maintaining a certain level of performance. Among the remaining 9 radiomic features, there is one original shape feature, three features derived from the Gaussian Laplacian transform (GLCM = 2; GLDM = 1), and five features obtained from wavelet transform (First order = 3; GLCM = 1; NGTDM = 1). The ranking of weights represented by the coefficients of each feature is shown in Supplementary Figure 3C.

The LASSO was also used to filter all extracted subregion features (Supplementary Figure 4A, B). Among the final 5 remaining features, 3 were from Subregion 3 (First order = 2; NGTDM = 1), and 2 were from Subregion 4 (Shape = 2). The weight images of each feature are shown in Supplementary Figure 4C.

### Deep transfer learning feature selection and modelling

After reducing the features extracted from the 2D and 3DLMs to 32, LASSO was used for further selection. Ultimately, the 2DLM retained 7 features, while the 3DLM retained 3 features. The LASSO feature selection process and the ranking of feature importance are shown Supplementary Figure 5. The 2D and 3D deep learning features were combined in various ways with other features to establish new models.

### Interpretability and stability of the models

In order to improve the interpretability of the model, gradient-weighted class activation mapping (Grad-CAM) was used to visualise the best model to generate the class activation map, which visually displayed the important regions that affected the decision making of the model. In addition, SHapley Additive exPlanations (SHAP) is a unified framework of different methods for interpreting predictions. The SHAP defines a class of feature importance measures and theoretical results that help illustrate the importance of features and their impact on the overall prediction model, and understanding the importance of individual features to model output [25]. We used SHAP to gain further insight into the characteristics of the best model.

In this study, the performance stability of the best model was evaluated in subgroups of age, gender, and CT scan thickness. From September 5, 2020 to October 11, 2020, we prospectively collected the imaging data of 18 patients with thymic tumours who underwent plain and enhanced chest CT scans. Radiomics, deep learning, clinical-visual imaging features and sub-regional features were extracted from the image data, and the results were predicted according to the best established model. The patient underwent resection of the anterior mediastinal mass within 10 days after the imaging examination, and the intraoperative specimen was examined pathologically. Pathological results were obtained after model prediction was completed. Furthermore, to validate the potential value of the model in clinical practice, we invited a junior doctor and a senior radiologist to independently predict thymoma grade with or without the assistance of the model. The work flow chart of this study is shown in Figure 2.

### Statistical analysis

The chi-square test or Fisher’s exact test was used to compare categorical variables, and the Mann-Whitney U test or independent *t*-test was used for continuous variables, with *p* values < 0.05 indicating statistical significance. The receiver operating characteristic curve (ROC) was drawn to evaluate the performance of each model, and the sensitivity, specificity, accuracy, positive predictive value, negative predictive value, and area under the curve (AUC) were calculated as evaluation indicators. Delong test was used to compare the differences in AUC among the models. The Calibration curve was drawn to evaluate the reliability and validity of the model. Decision Curve Analysis (DCA) was used to evaluate the clinical usefulness of the model. All statistical analysis were performed with the use of Python (version 3.9.12) or R (version 4.2.1).

## Results

### Patient characteristics

As shown in Table 1, the results of univariate analysis of baseline clinical and demographic data showed that in the training cohort, there were no significant differences in gender and age between the LRT group and the HRT group (*p* = 0.879; *p* = 0.078). In training cohort, the following conventional imaging features did not show statistical differences in univariate analysis between HRT and LRT: lobulated, primary site, calcification, boundary clarity, myasthenia gravis, thoracic disease, symptoms, 2D maximum axis of tumour, pre-contrast CT attenuation in HU, post-contrast CT attenuation in HU, and change of CT value in HU values. Compared with the HRT group, the LRT group tended to have more uniform density and more regular shape (*p* = 0.047, 0.042). In addition, the 3D maximum diameter (51.69±25.65) was larger in the LRT group (*p* = 0.037). Multivariate analysis of density uniformity, shape, and 3D maximum diameter (Supplementary Table 3) showed that there were statistical differences in the three variables, which were determined to be independent predictors of thymoma risk stratification. This was used together with tumour subregion features to construct the CSM.

**Table 1:** Characteristic baseline.

Characteristics	Training cohort (*n* = 100)			Internal validation cohort (*n* = 44)			External validation cohort1 (*n* = 44)			External validation cohort2 (*n* = 17)		
	High risk (*n* = 41)	Low risk (*n* = 59)	*P*	High risk (*n* = 17)	Low risk (*n* = 27)	*P*	High risk (*n* = 17)	Low risk (*n* = 27)	*P*	High risk (*n* = 9)	Low risk (*n* = 8)	*P*
Age, median (range) (years)	52(30–77)	57(15–81)	0.078	55(18–72)	57(19–74)	0.755	51(37–69)	52(36–72)	0.82	54(31–59)	50(48–71)	0.228
Sex, *n* (%)			0.879			0.109			0.907			0.457
Male	23 (56%)	34 (58%)		14 (82%)	16 (59%)		11 (65%)	17 (63%)		4 (44%)	5 (63%)	
Female	18 (44%)	25 (42%)		3 (18%)	11 (41%)		7 (35%)	10 (37%)		5 (56%)	3 (37%)	
Lobulated, *n* (%)			0.526			0.757			0.017*			0.707
Present	20 (49%)	25 (42%)		8 (47%)	14 (52%)		31 (69%)	17 (74%)		3 (33%)	2 (25%)	
Absent	21 (51%)	34 (58%)		9 (52%)	13 (48%)		14 (31%)	6 (26%)		6 (67%)	6 (75%)	
Primary site, *n* (%)			0.159			0.651			0.043*			0.858
Side	26 (63%)	29 (49%)		7 (41%)	13 (48%)		8 (47%)	5 (19%)		3 (33%)	3 (37%)	
Middle	15 (37%)	30 (51%)		10 (59%)	14 (52%)		9 (53%)	22 (81%)		6 (67%)	5 (63%)	
Density uniformity, *n* (%)			0.047*			0.718			0.242			0.086
Uniform	14 (34%)	32 (54%)		6 (35%)	11 (41%)		10 (59%)	11 (41%)		3 (33%)	6 (75%)	
Non-uniform	27 (66%)	27 (46%)		11 (65%)	16 (59%)		7 (41%)	16 (59%)		6 (67%)	2 (25%)	
Shape, *n* (%)			0.042*			0.055			0.784			0.457
Regular	18 (44%)	38 (64%)		7 (41%)	19 (70%)		10 (59%)	17 (63%)		5 (56%)	3 (37%)	
Irregular	23 (56%)	21 (36%)		10 (59%)	8 (30%)		7 (41%)	10 (37%)		4 (44%)	5 (63%)	
Calcification, *n* (%)			0.056			0.538			0.92			0.149
Present	10 (24%)	6 (10%)		3 (18%)	3 (11%)		4 (24%)	6 (22%)		4 (44%)	1 (13%)	
Absent	31 (76%)	53 (90%)		14 (82%)	24 (89%)		13 (76%)	21 (78%)		5 (56%)	7 (87%)	
Boundary clarity, *n* (%)			0.997			0.68			0.363			0.149
Clear	25 (61%)	36 (61%)		9 (53%)	16 (59%)		9 (53%)	18 (67%)		5 (56%)	7 (87%)	
Vague	16 (39%)	23 (39%)		8 (47%)	11 (41%)		8 (47%)	9 (33%)		4 (44%)	1 (13%)	
Myasthenia gravis, *n* (%)			0.106			0.92			0.774			0.453
Present	8 (20%)	5 (8%)		4 (24%)	6 (22%)		2 (12%)	4 (15%)		1 (11%)	2 (25%)	
Absent	33 (80%)	54 (92%)		13 (76%)	21 (78%)		15 (88%)	23 (85%)		8 (89%)	6 (75%)	
Thoracic disease, *n* (%)			0.31			0.251			0.155			0.929
Present	8 (20%)	8 (14%)		4 (24%)	2 (7%)		0 (0%)	3 (11%)		1 (11%)	1 (13%)	
Absent	33 (80%)	51 (86%)		13 (76%)	25 (93%)		17 (100%)	24 (89%)		8 (89%)	7 (87%)	
Symptoms, *n* (%)			0.74			0.624			0.363			0.453
Present	4 (10%)	7 (12%)		2 (12%)	2 (7%)		1 (6%)	4 (15%)		1 (11%)	2 (25%)	
Absent	37 (90%)	52 (88%)		15 (88%)	25 (93%)		16 (94%)	23 (85%)		8 (89%)	6 (75%)	
2D maximum axis diameter (mean ± SD) in mm	42.51 ± 22.45	44.3 ± 19.34	0.126	47.79 ± 27.53	33.75 ± 2.76	0.319	48.12 ± 15.42	54.95 ± 20.83	0.25	41.92 ± 16.14	27.87 ± 11.38	0.059
3D maximum diameter (mean ± SD) in mm	47.35 ± 22.14	51.69 ± 25.65	0.037*	53.90 ± 32.26	45.99 ± 13.00	0.632	53.33 ± 16.12	62.86 ± 27.28	0.153	48.71 ± 23.65	30.67 ± 13.84	0.079
Pre-contrast CT attenuation (mean ± SD) in HU	31.06 ± 9.03	32.14 ± 10.91	0.597	34.31 ± 11.05	26.15 ± 7.63	0.159	42.24 ± 9.04	42.38 ± 11.56	0.968	23.12 ± 8.68	22.67 ± 9.80	0.922
Post-contrast CT attenuation (mean ± SD) in HU	60.39 ± 11.90	64.31 ± 16.78	0.136	64.69 ± 19.35	53.91 ± 4.45	0.277	74.46 ± 13.48	77.77 ± 22.55	0.589	61.77 ± 13.22	66.48 ± 16.85	0.528
Change of CT value (mean ± SD) in HU	29.33 ± 10.75	32.17 ± 13.66	0.477	30.38 ± 15.16	27.76 ± 5.04	0.735	32.22 ± 11.16	35.39 ± 13.60	0.425	38.65 ± 11.32	43.81 ± 14.98	0.433

*P* < 0.05, significant difference, Use * tag. Differences were compared using the *t* test; Mann–Whitney *U* test; Chi-square test or Fisher’s exact test.

Continuous variables: represented as mean ± standard deviation (SD); Categorical variable: represented as [number and percentage (%)].

CT: computed tomography; HU: Hounsfield unit; Change of CT value = Post-contrast CT attenuation− Pre-contrast CT attenuation.

### Auto-segmentation performance

The Deeplabv3 segmentation model developed by us has an average Loss, Dice score, IoU, precision, and recall of 0.147, 0.762, 0.836, 0.820 and 0.912 in the test dataset over 50 epochs, respectively. Lesion segmentation results and model training process are shown in Supplementary Figure 2.

### Performance evaluation and comparison of models

The diagnostic metrics and ROCs of all individual feature models that were not integrated are summarised in the training cohort, internal validation cohort, and two external validation cohorts; and are presented in Supplementary Figures 6, 7, and 8, and Table 2. In the RM, the classifier LightGBM achieved the highest AUC in the training, internal validation, and external validation cohort 1, with values of 0.847 (95% CI: 0.771–0.923), 0.779 (95% CI: 0.633–0.925), and 0.788 (95% CI: 0.645–0.930), respectively. In external validation cohort 1, the accuracy (ACC), sensitivity (SEN), specificity (SPE), positive predictive value (PPV), and negative predictive value (NPV) were 0.727, 0.647, 0.778, 0.647, and 0.778, respectively; all of which were superior to kNN and MLP. Resnet152 had the highest AUC in the training cohort, internal validation cohort, and external validation cohort 2 for the 2DLM, with values of 0.849 (95% CI: 0.774–0.925), 0.731 (95% CI: 0.577–0.885), and 0.777 (95% CI: 0.533–1.000), respectively. Resnet152 was selected as the representative model of 2DLM for subsequent feature fusion. Mobilenet_v2 had the highest AUC in external validation cohort 1 of the 2DLM, with a value of 0.784 (95% CI: 0.618–0.951). Compared to the 2DLM, the 3DLM exhibited a better AUC in the internal validation cohort (0.702–0.796 vs. 0.643–0.731), but no significant differences were observed in the other data cohorts. Among the 3DLMs, Resnet18 demonstrated the best performance, with an AUC of 0.750–0.875, and was selected as the representative model for further feature integration.

**Table 2:** The performance of various unfused models.

Model	Training cohort						Internal-validation cohort						External-validation cohort1						External-validation cohort2					
	AUC (95% CI)	ACC	SEN	SPE	PPV	NPV	AUC (95% CI)	ACC	SEN	SPE	PPV	NPV	AUC (95% CI)	ACC	SEN	SPE	PPV	NPV	AUC (95% CI)	ACC	SEN	SPE	PPV	NPV
**RM**																								
kNN	0.846 (0.773–0.920)	0.770	0.439	1.000	1.000	0.720	0.716 (0.571–0.861)	0.636	0.471	0.741	0.533	0.690	0.706 (0.557–0.855)	0.636	0.529	0.704	0.529	0.704	0.722 (0.462–0.983)	0.588	0.333	0.875	0.750	0.538
**LightGBM**	0.847 (0.771–0.923)	0.810	0.732	0.864	0.789	0.823	0.779 (0.633–0.925)	0.705	0.706	0.704	0.600	0.792	0.788 (0.645–0.930)	0.727	0.647	0.778	0.647	0.778	0.688 (0.421–0.954)	0.647	0.444	0.875	0.800	0.583
MLP	0.811 (0.726–0.895)	0.750	0.610	0.847	0.735	0.758	0.763 (0.619–0.907)	0.727	0.647	0.778	0.647	0.778	0.702 (0.540–0.863)	0.682	0.647	0.704	0.579	0.760	0.764 (0.522–1.000)	0.706	0.667	0.750	0.750	0.667
**2DLM**																								
Densenet161	0.834 (0.749–0.921)	0.790	0.810	0.776	0.723	0.849	0.724 (0.574–0.875)	0.659	0.882	0.519	0.536	0.875	0.706 (0.534–0.888)	0.773	0.588	0.889	0.769	0.774	0.722 (0.448–0.996)	0.706	0.556	0.875	0.833	0.636
GoogLeNet	0.757 (0.663–0.852)	0.720	0.619	0.793	0.684	0.742	0.643 (0.466–0.820)	0.636	0.706	0.593	0.522	0.762	0.610 (0.425–0.795)	0.614	0.647	0.593	0.500	0.727	0.681 (0.404–0.957)	0.647	0.667	0.625	0.667	0.625
Mobilenet_v2	0.786 (0.686–0.886)	0.810	0.667	0.914	0.848	0.791	0.675 (0.509–0.842)	0.682	0.529	0.778	0.600	0.724	0.784 (0.618–0.951)	0.841	0.706	0.926	0.857	0.833	0.722 (0.458–0.987)	0.706	0.556	0.875	0.833	0.636
**Resnet152**	0.849 (0.774–0.925)	0.780	0.881	0.707	0.685	0.891	0.731 (0.577–0.885)	0.636	0.824	0.519	0.519	0.824	0.753 (0.594–0.911)	0.773	0.588	0.889	0.769	0.774	0.777 (0.533–1.000)	0.706	0.556	0.875	0.833	0.636
**3DLM**																								
Densenet201	0.726 (0.627–0.824)	0.690	0.690	0.690	0.617	0.755	0.708 (0.546–0.870)	0.659	0.765	0.593	0.542	0.800	0.654 (0.477–0.830)	0.614	0.765	0.519	0.500	0.778	0.653 (0.362–0.944)	0.647	0.556	0.750	0.714	0.600
**Resnet18**	0.875 (0.809–0.942)	0.780	0.881	0.707	0.685	0.891	0.796 (0.654–0.939)	0.750	0.706	0.778	0.667	0.808	0.763 (0.607–0.920)	0.727	0.706	0.741	0.632	0.800	0.805 (0.551–1.000)	0.824	0.667	1.000	1.000	0.727
ShuffleNet	0.829 (0.733–0.925)	0.870	0.762	0.948	0.914	0.846	0.753 (0.598–0.908)	0.773	0.412	1.000	1.000	0.730	0.721 (0.552–0.891)	0.773	0.471	0.963	0.889	0.743	0.708 (0.427–0.989)	0.706	0.556	0.875	0.833	0.636
ViT	0.822 (0.735–0.910)	0.800	0.738	0.845	0.775	0.817	0.702 (0.525–0.879)	0.750	0.471	0.926	0.800	0.735	0.682 (0.510–0.854)	0.727	0.529	0.852	0.692	0.742	0.694 (0.417–0.972)	0.706	0.556	0.875	0.833	0.636

RM: radiomic model; 2DLM: 2D deep learning; 3DLM: 3D deep learning; AUC: area under the summary receiver operating characteristic curve; ACC: accuracy; SEN: sensitivity; SPE: specificity; CI: confidence interval; PPV: positive predictive value; NPV: negative predictive value; kNN: K-Nearest Neighbor; LightGBM: Light Gradient Boosting Machine; MLP: Multilayer Perceptron; ViT: vision transformer.

The name is indicated in bold as the best model and can be used as a representative model. For example, LightGBM is a representative model for RM.

To explore whether different types of features have a synergistic effect on predicting thymoma risk stratification, we developed two-types feature fusion models (R2DLM, R3DLM, DLM, CSM), three-types feature fusion models (RDLM, RCSM, 2DLCSM, 3DLCSM), and five-types feature fusion models (RDLCSM) through individual feature pre-fusion. The summary of various predictive metrics is shown in Table 3.

**Table 3:** The performance comparison of different models.

Model (Classifier)	Training cohort						Internal-validation cohort						External-validation cohort1						External-validation cohort2					
	AUC (95% CI)	ACC	SEN	SPE	PPV	NPV	AUC (95% CI)	ACC	SEN	SPE	PPV	NPV	AUC (95% CI)	ACC	SEN	SPE	PPV	NPV	AUC (95% CI)	ACC	SEN	SPE	PPV	NPV
**Two types of feature fusion model**																								
R2DLM (MLP)	0.833 (0.751–0.916)	0.800	0.634	0.915	0.839	0.783	0.784 (0.636–0.933)	0.727	0.412	0.926	0.778	0.714	0.719 (0.552–0.886)	0.682	0.353	0.889	0.667	0.686	0.819 (0.615–1.000)	0.706	0.556	0.875	0.833	0.636
R3DLM (LightGBM)	0.836 (0.756–0.916)	0.790	0.634	0.898	0.813	0.779	0.778 (0.627–0.929)	0.727	0.471	0.889	0.727	0.727	0.760 (0.615–0.905)	0.705	0.529	0.815	0.643	0.733	0.722 (0.455–0.990)	0.765	0.667	0.875	0.857	0.700
DLM (LightGBM)	0.867 (0.792–0.943)	0.750	0.561	0.881	0.767	0.743	0.725 (0.561–0.890)	0.750	0.529	0.889	0.750	0.750	0.743 (0.575–0.910)	0.682	0.235	0.963	0.800	0.667	0.819 (0.574–1.000)	0.706	0.556	0.875	0.833	0.636
CSM (SVM)	0.798 (0.708–0.887)	0.670	0.366	0.881	0.682	0.667	0.736 (0.581–0.892)	0.659	0.235	0.926	0.667	0.658	0.725 (0.567–0.884)	0.614	0.235	0.852	0.500	0.639	0.708 (0.431–0.985)	0.765	0.778	0.750	0.778	0.750
**Three types of feature fusion model**																								
RDLM (kNN)	0.890 (0.831–0.950)	0.810	0.585	0.966	0.923	0.770	0.831 (0.712–0.951)	0.773	0.647	0.852	0.733	0.793	0.871 (0.771–0.972)	0.773	0.706	0.815	0.706	0.815	0.847 (0.662–1.000)	0.824	0.778	0.875	0.875	0.778
RCSM (ET)	0.887 (0.826–0.949)	0.880	0.927	0.847	0.809	0.943	0.830 (0.710–0.950)	0.750	0.765	0.741	0.650	0.833	0.808 (0.682–0.935)	0.750	0.588	0.852	0.714	0.767	0.792 (0.556–1.000)	0.706	0.667	0.750	0.750	0.667
2DLCSM (kNN)	0.916 (0.865–0.967)	0.820	0.805	0.831	0.767	0.860	0.876 (0.759–0.992)	0.818	0.882	0.778	0.714	0.913	0.814 (0.682–0.946)	0.727	0.529	0.852	0.692	0.742	0.785 (0.557–1.000)	0.706	0.556	0.875	0.833	0.636
3DLCSM (kNN)	0.842 (0.769–0.915)	0.770	0.780	0.763	0.696	0.833	0.768 (0.621–0.915)	0.727	0.647	0.778	0.647	0.778	0.746 (0.605–0.887)	0.727	0.588	0.815	0.667	0.759	0.778 (0.557–0.998)	0.765	0.667	0.875	0.857	0.700
**Five types of feature fusion model**																								
RDLCSM (LightGBM)	0.953 (0.906–0.999)	0.880	0.756	0.966	0.939	0.851	0.930 (0.854–1.000)	0.818	0.647	0.926	0.846	0.806	0.924 (0.849–0.999)	0.841	0.647	0.963	0.917	0.813	0.903 (0.710–1.000)	0.882	0.778	1.000	1.000	0.800

AUC: area under the summary receiver operating characteristic curve; ACC: accuracy; SEN: sensitivity; SPE: specificity; CI: confidence interval; PPV: positive predictive value; NPV: negative predictive value; R2DLM: radiomics + 2D deep learning; R3DLM: radiomics + 3D deep learning; DLM: 2D deep learning + 3D deep learning; CSM: clinical-visual radiological + tumour subregion; RDLM: radiomics + 2D deep learning + 3D deep learning; RCSM: radiomics + clinical-visual radiological + tumour subregion; 2DLCSM: 2D deep learning + clinical-visual radiological + tumour subregion; 3DLCSM: 3D deep learning + clinical-visual radiological + tumour subregion; RDLCSM: radiomics + 2D deep learning + 3D deep learning + clinical-visual radiological + tumour subregion; RDLCSM: radiomics + 2D deep learning + 3D deep learning + clinical-visual radiological + tumour subregion; KNN: K-Nearest Neighbor; LightGBM: Light Gradient Boosting Machine; MLP: Multilayer Perceptron; SVM: Support vector Machine; ET: Extra trees.

For the two types of feature fusion models, the AUC ranges for R2DLM, R3DLM, DLM, and CSM across data cohorts were 0.719–0.833, 0.722–0.836, 0.725–0.867, and 0.708–0.798, respectively. In comparison, the DLM model based on the LightGBM classifier showed better performance and achieved the highest accuracy (ACC: 0.750), sensitivity (SEN: 0.529), and negative predictive value (NPV: 0.750) in the internal validation cohort, while its specificity (SPE) and positive predictive value (PPV) in external validation cohort 2 were 0.963 and 0.800, respectively. However, the Delong test results indicated that the predictive performances of the four two-types feature fusion models showed no significant differences across the training cohort, internal validation cohort, and two external validation cohorts (*p*-values ranging from 0.189 to 0.963) (Supplementary Figure 9).

**Figure 3:**
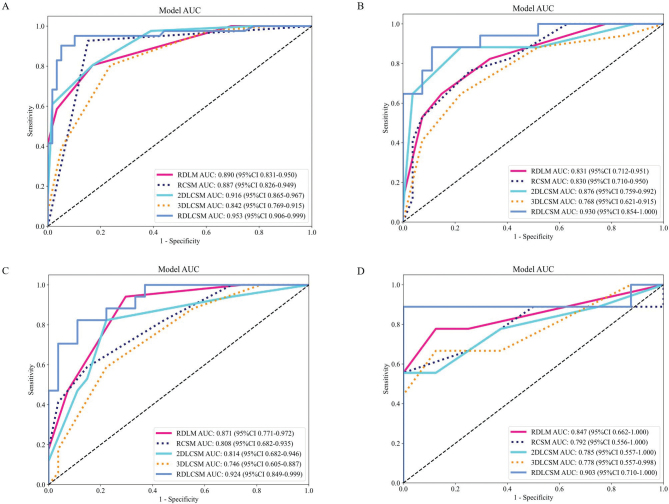
ROC curves of three and five types of feature fusion models. The ROC curves of three and five types of feature fusion models in the training cohort (A), internal validation cohort (B), external validation cohort1 (C), and external validation cohort2 (D). AUC: area under the summary receiver operating characteristic curve; CI: confidence interval. Training cohort (*n* = 100); Internal validation cohort (*n* = 44); External validation cohort1 (*n* = 44); External validation cohort2 (*n* = 17).

**Figure 4:**
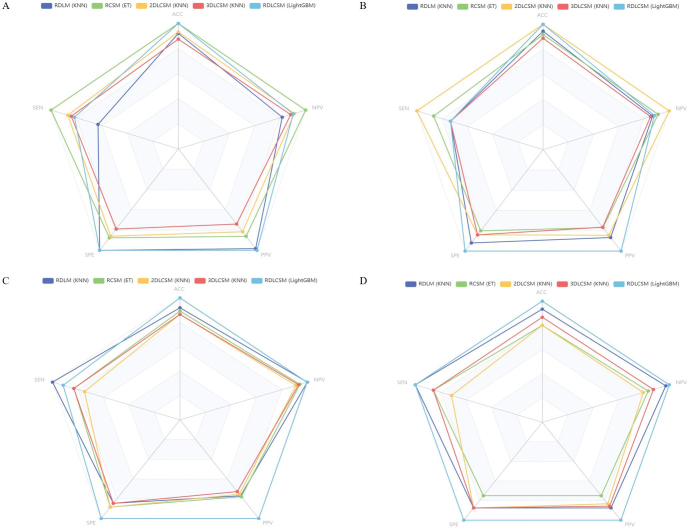
Radar plot of three and five types of feature fusion models. The radar plot of three and five types of feature fusion models in the training cohort (A), internal validation cohort (B), external validation cohort1 (C), and external validation cohort2 (D).

In the three types of feature fusion models, the AUC ranges for RDLM, RCSM, 2DLCSM, and 3DLCSM across data cohorts were 0.831–0.890, 0.792–0.887, 0.785–0.916, and 0.746–0.842, respectively (Figure 3). Both RDLM and 2DLCSM, built on the kNN classifier, had the highest and second highest AUC values in the training cohort, internal validation cohort, and external validation cohort 1, but the AUC for 2DLCSM in the external validation cohort was 0.785 (95% CI: 0.557–1.000), which was lower than that of 3DLCSM (0.778; 95% CI: 0.557–0.998) and RCSM (0.792; 95% CI: 0.556–1.000). When comparing the three-types and five-types feature fusion models, the results showed that in the training cohort, 3DLCSM performed worse than 2DLCSM (*p*-value: 0.027). Furthermore, the three types of feature fusion model RDLCSM, based on LightGBM, achieved the highest AUC across all cohorts (0.903–0.953) and outperformed the 3DLCSM model in the training cohort, internal validation cohort, and external validation cohort 1 (*p*-values of 0.003, 0.014, and 0.01, respectively). Other metrics can be compared in Figure 4. A summary of fusion model characteristics is provided in the Supplementary Table 4.

The calibration curves indicate (Supplementary Figure 10) that compared to RDLM, 2DLCSM, and RDLCSM, RDLM has the best goodness of fit and relatively high prediction probability accuracy across all data cohorts. The RDLCSM demonstrates better model fit than 2DLCSM in the internal validation cohort, but not in the training cohort, where 2DLCSM outperformed RDLCSM; the model fitting performance of both in the external validation cohorts is somewhat similar. In DCA, RDLCSM maintained a high net benefit across different data cohorts, particularly evident at risk thresholds between 0.4 and 0.6 (Supplementary Figure 11).

### Models stability and practicability assessment

To explore the impact of different variables on the predictive performance of the models, we conducted a stratified analysis of the RDLM, 2DLCSM, and RDLCSM models based on three common factors: gender, age (with a median of 54 in the overall dataset), and the thickness of CT scan layers (Supplementary Figure 12). Overall, gender (male or female) did not show a significant effect on the three models. Both RDLM and 2DLCSM maintained good consistency across different age groups (>54 years or <54 years), while the RDLCSM model achieved an AUC of 0.912 in the subgroup aged <54 years, which was higher than the AUC values of the other models in the age subgroups; however, the DeLong test showed no statistical difference. The AUC ranges for the three models in different layer thickness subgroups (0.6 mm, 1 mm, 1.25 mm) were 0.790–0.919 for RDLM, 0.752–0.854 for 2DLCSM, and 0.864–0.904 for RDLCSM, with RDLCSM demonstrating the best performance.

Among the 18 patients collected in the prospective trial, the final pathological results showed 7 cases of HRT and 11 cases of LRT. Before knowing the pathological results, the RDLM, 2DLCSM, and RDLCSM models were used to predict the risk stratification of thymoma. The RDLCSM correctly predicted all 7 HRT patients, while RDLM and 2DLCSM correctly predicted 6. Among the 11 LRT patients, both RDLCSM and RDLM correctly predicted 9, while 2DLCSM correctly predicted 6. The predictive performance metrics are summarised in Supplementary Table 5, where the AUCs for RDLM, 2DLCSM, and RDLCSM were 0.825 (95% CI: 0.588–1.000), 0.792 (95% CI: 0.542–1.000), and 0.909 (95% CI: 0.771–1.000), respectively, with RDLCSM achieving the highest values in all metrics except for specificity.

Supplementary Figure 13 and Supplementary Table 6 summarise the diagnostic performance of radiologists in thymoma risk stratification with and without the assistance of RDLM, 2DLCSM, and RDLCSM. The results indicated that the overall performance of junior radiologists significantly improved with model assistance, with AUC increasing by 0.112–0.192, ACC improving by 0.117–0.195, SEN increasing by 0.141–0.207, PPV rising by 0.142–0.233, and NPV increasing by 0.083–0.152. For senior radiologists, predictive performance also showed enhancement with model assistance. Junior radiologists were able to reach or even surpass the predictive capabilities of senior radiologists without model assistance.

### Models interpretability analysis

**Figure 5:**
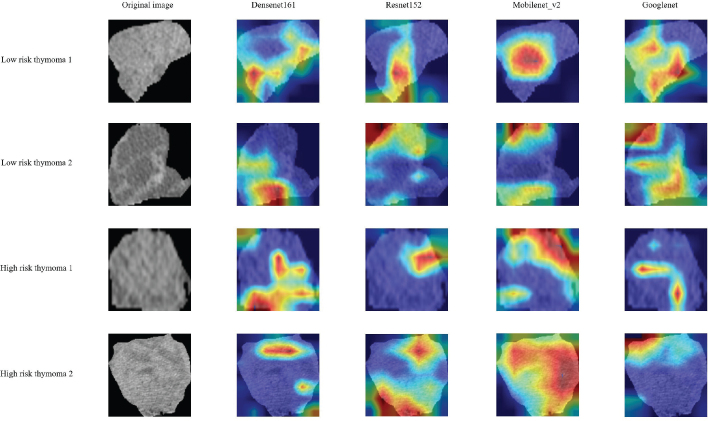
The attention regions of different deep learning models. We selected images from two LRT and two HRT patients. Grad-CAM was used for deep learning model visualization. The closer the colour of the attention area is to red, the greater the weight is.

Figure 5 shows the coloured heat maps generated by four different 2DLM models, revealing that various convolutional neural networks exhibit attention areas across different parts of thymoma, rather than tending to focus solely on the tumour’s centre or edges. However, whether HRT or LRT, multiple attention areas exist in most cases, which may have some correlation with the heterogeneity of internal tumour subregions.

**Figure 6:**
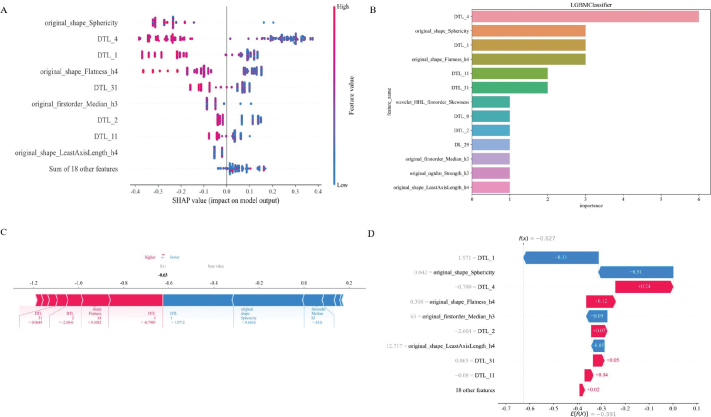
SHAP visually interprets machine learning models. Figures A, C, and D represent SHAP_summary plot, SHAP_force plot, and SHAP_waterfall plot, respectively. Figure B represents the feature importance ranking map based on the RDLCSM generated by LightGBM. DTL and DL are used for prefix naming of 2D and 3D deep learning features, respectively. For example, DTL_27 is the 28th 2D deep learning feature after PCA (the first one is named 0), and similarly, DL_29 represents the 30th 3D deep learning feature after PCA. SHAP, SHapley Additive exPlanations.

We used SHAP to provide a quantitative interpretation of the RDLCSM. The SHAP summary plot calculates the SHAP values for each patient’s corresponding features and ranks these features based on their importance to the overall model. In Figure 6A, it can be seen that original_shape_Sphericity is the most important feature; when the value of original_shape_Sphericity is low, the model is more likely to output a result of LRT, which is consistent with the feature coefficients obtained from LASSO in Supplementary Figure 3C. Since the RDLCSM is constructed using the LightGBM classifier, we further presented its feature weight chart (Figure 6B), where deep transfer learning (DTL_4) and original_shape_Sphericity are regarded as the first and second most important features, although the order differs from that in Figure 6A, both features are still considered the two most significant. Furthermore, both Figures 6A and 6B identify DTL_1 and original_shape_Flatness_h4 as the third and fourth most important features, respectively. Figure 6C displays a SHAP force plot, which explains the evaluation process for an individual patient. As shown in Figure 6C, the patient’s SHAP value is –0.63, which is less than the base value of –0.391, leading to a predicted result of LRT that is consistent with the pathological findings. Above each feature, there is an arrow; red indicates a positive contribution, while blue represents a negative contribution, with the arrow’s length indicating the strength of the feature’s contribution. From the figure, it is evident that DTL_1 and original_shape_Sphericity make significant contributions to the evaluation of LRT. Figure 6D provides a SHAP waterfall chart for easy comparison of the specific contribution levels of each feature.

## Discussion and conclusion

Almost all incidental thymic tumours are asymptomatic and lack appropriate serum markers; therefore, the clinician’s decision on treatment largely depends on imaging findings [26]. Histological features influence tumour behaviour and prognosis, and accurate classification of thymoma by medical imaging is essential in clinical practice [27]. Conventional CT and MRI can provide detailed morphological information about tumour size, shape, homogeneity, and other features, but they cannot correctly distinguish the pathological classification and stage of thymoma [28]. In this study, we used radiomics, multi-dimensional deep learning, clinical-visual radiological and habitat analysis based on contrast-enhanced CT to predict thymoma risk stratification.

After screening the extracted radiomics features based on the whole tumour, among the 9 retained radiomics features, 3 were GLCM. On the one hand, GLCM features accounted for a large proportion of the original features; on the other hand, reflecting the strong spatial correlation of the grey levels of each voxel in thymoma images with different risk classifications. This is consistent with the findings of Li et al. [29]. The tumours were divided into multiple subregions by K-means clustering method, and the corresponding features were extracted. The five retained features were all from subregions 3 and 4, indicating that these two regions were highly heterogeneous in LRT and HRT. Comprehensive analysis of the features of the whole tumour and subregions showed that the first-order features (Skewness, Median) accounted for a high proportion. In addition, the feature weight plots in Supplementary Figure 3 and Supplementary Figure 4 showed that ngtdm_Strength was the feature with the largest positive weight both in the whole tumour and in the subregion features. The ngtdm is not limited by image size and resolution and is suitable for all types of images. Moreover, the operation speed is fast, and it is suitable for the processing and analysis of large-scale image data. It shows that ngtdm_Strength derived from wavelet transform is of great significance for the differentiation of thymoma with different risks. The features with the largest negative weight were all shape features (Sphericity and Flatness), indicating that the more spherical the lesion was, the flatness was more likely to be LRT, which was correlated with the results of multivariate analysis of clinical-visual radiological features. It has been shown that there is no difference in size between thymoma subtypes, which may be due to the fact that they are only measured on axial images [30]. We measured the maximum length of the tumour by 3D reconstruction, and the results showed that LRT tended to have a larger length in the training cohort, which may suggest that this parameter has some value.

At present, only a few studies have used deep learning methods to explore the risk stratification of thymoma. Liu et al. [18] used Vgg19 to construct a model with a sample size of 150 cases in a single centre, which achieved good model performance, but its generalisation ability in other medical centres is unknown. The constructed radiomics and deep learning models used only one classifier or convolutional neural network. The 3D_Resnet50 model they developed in their later study also demonstrated the advantages of deep learning. However, there is a lack of exploration on the integration of multiple models, and the interpretability of models is poor [31]. We built 2D (Densenet161, Googlenet, Mobilenet_v2, Resnet152) and 3D (Densenet201, Resnet18, ShuffleNet) deep learning models through multiple convolutional neural networks. And we introduced ViT to compare with the traditional convolutional neural network. The results showed that Resnet152 and Resnet18 achieved the best performance in the 2D and 3DLMs, with AUC ranges of 0.731–0.849 and 0.763–0.875, respectively. However, 2D and 3DLMs do not show significant performance differences in each data cohort, which may be due to the fact that 3DLMs often require a large amount of data to effectively learn complex spatial features, otherwise it often leads to poor model performance or overfitting [24].

Differences in factors such as cell density, proliferation rate, angiogenesis, hypoxia and the proportion of necrosis within tumours can cause tumour heterogeneity. The pathop-hysiological basis of tumour aggressiveness is complex and often involves multiple mechanisms, and the exact relationship between pathological findings and higher-order features remains to be fully elucidated [32]. We combined the features of subregions with whole-tumour radiomics, deep learning models, and clinical-visual radiological features to develop two-types, three-types, and five-types feature fusion models. The model performance, fit degree, and clinical benefit were compared. After comprehensive evaluation, RDLM, 2DLCSM, and RDLCSM had good overall performance. Prospective trials, stratified analysis, and radiologist assistance were used to further compare the three models. With the assistance of the three models, the diagnostic performance of junior and senior radiologists was improved. Stratified analysis showed that age, gender, and CT slice thickness had no significant effect on the performance of the model, and RDLM, 2DLCSM and RDLCSM had good stability. In the prospective study, RDLCSM correctly predicted the largest number of patients with an AUC of 0.909 (95% CI:0.771–1.000). In general, RDLCSM has the best performance, excellent stability, and excellent generalisation ability; and can be used as the first choice for prediction model. However, this model contains too many parameters and is time-consuming in clinical practice. If simplicity is more important, you should choose RDLM or 2DLCSM.

Our study still has certain limitations. Firstly, the study was a retrospective study overall, and the sample size of the prospective study was too small; more prospective centre cases are needed to confirm our findings. Secondly, variations in scanning equipment and protocols as well as contrast media may lead to additional bias in extracting features. Thirdly, because deep learning is derived from dimensionality reduction through PCA compression, each feature cannot have a specific meaning like radiomics features. Therefore, we used SHAP to further understand it and try to find its practical application value in all patients and individual patients. We will further explore the potential associations between radiomics features and deep learning features in future studies. Finally, all the models in this study were only based on enhanced CT images in the venous phase, and the plain scan and other phases were ignored. Considering that tumours may show different performance at different time stages, we will collect data in different enhancement stages and compare the performance of corresponding models in future studies.

## Conclusion

In conclusion, the whole-tumour radiomics, multi-dimensional deep learning, clinical-visual radiology, and biogenic subregion fusion model developed in this study can accurately predict thymoma risk classification based on preoperative contrast-enhanced CT image data, which can improve the diagnostic ability of radiologists with different levels of experience and assist clinicians to make informed and personalised medical decisions.

## Supplementary Material



## Data Availability

The data that support the findings of this study are available on request from the corresponding author, upon reasonable request.
