# Supplementary material for: Multi-dimensional interpretable deep learning-radiomics based on intra-tumoral and spatial habitat for preoperative prediction of thymic epithelial tumours risk categorisation
Source: Acta Oncol. 2025 Mar 13;64:42982. doi: 10.2340/1651-226X.2025.42982 (PMC11971837; doi:10.2340/1651-226X.2025.42982)

Supplementary material has been published as submitted. It has not been copyedited, or typeset by Acta Oncologica

Supplementary Table 1: Relevant parameters of the CT equipment used

	GE Revolution CT	SOMATOM Definition Flash	Philips 256 iCT	SOMATOM Definition Flash	GE Light Speed VCT	GE Revolution CT	SOMATOM Definition AS Flash	SOMATOM Definition Flash
CT tube voltage	120 kVp	120 kVp	120 kVp	120 kVp	120 kVp	120 kVp	120 kVp	120 kVp
CT tube current	Adaptive	150mA	150mA	Adaptive	200mA	Adaptive	Adaptive	Adaptive
CT rotation time	0.75s	0.5s	0.5s	0.5s	0.6S	0.5s	0.5s	0.5s
Pitch	0.984	1	0.758	1	1.375	0.986	0.952	1
Layer thickness	5mm	5mm	5mm	5mm	5mm	5mm	5mm	5mm
Reconstruction thickness	1.25mm	1mm	1mm	0.6mm	1.25mm	1mm	0.6mm	1mm
Reconstruction pitch	1.25mm	1mm	1mm	0.6mm	1.25mm	1mm	0.6mm	1mm
Image matrix	512*512	512*512	512*512	512*512	512*512	512*512	512*512	512*512
kernel	B50s	B50f	B80f	B70f	B51s	B51f	B51f	B80f
Reconstruction algorithm	B-SOFT-B	Standard Algorithm	Standard Algorithm	B-SOFT-B	B-SOFT-B	B-SOFT-B	B-SOFT-B	B-SOFT-B

Supplementary Table 2:K-means clustering index evaluation

Clusters（K value)	Calinski-Harabasz Index	Silhouette Coefficient	Davies-Bouldin Index
2	533372.731	0.430	0.868
3	558816.817	0.394	0.839
4	561580.096	0.360	0.800
5	523365.524	0.318	0.894

Supplementary Table 3：The results of multivariate analysis

Characteristics	Multivariate analysis			
	Regression coefficient	Odds ratio	95% confidence interval	P-value
Shape	1.135	3.112	1.187-8.156	0.021*
Density uniformity	1.134	3.109	1.196-8.079	0.02*
3D maximum diameter	-0.043	0.958	0.933-0.984	0.002*

P<0.05, significant difference.

Supplementary Table 4: Features of models

Model	Feature name								
**RM**	log_sigma_1_0_mm_3D_glcm_Imc2	log_sigma_1_0_mm_3D_glcm_InverseVariance	log_sigma_1_0_mm_3D_gldm_DependenceVariance	original_shape_Sphericity	wavelet_HHH_firstorder_Skewness	wavelet_HHL_firstorder_Skewness	wavelet_HLH_glcm_Imc1	wavelet_HLL_ngtdm_Strength	wavelet_LHL_firstorder_Median
**2DLM**	DTL_0	DTL_1	DTL_2	DTL_4	DTL_11	DTL_27	DTL_31		
**3DLM**	DL_9	DL_11	DL_29						
**R2DLM**	log_sigma_1_0_mm_3D_glcm_Imc2	log_sigma_1_0_mm_3D_glcm_InverseVariance	log_sigma_1_0_mm_3D_gldm_DependenceVariance	original_shape_Sphericity	wavelet_HHH_firstorder_Skewness	wavelet_HHL_firstorder_Skewness	wavelet_HLH_glcm_Imc1	wavelet_HLL_ngtdm_Strength	wavelet_LHL_firstorder_Median
	DTL_0	DTL_1	DTL_2	DTL_4	DTL_11	DTL_27	DTL_31		
**R3DLM**	log_sigma_1_0_mm_3D_glcm_Imc2	log_sigma_1_0_mm_3D_glcm_InverseVariance	log_sigma_1_0_mm_3D_gldm_DependenceVariance	original_shape_Sphericity	wavelet_HHH_firstorder_Skewness	wavelet_HHL_firstorder_Skewness	wavelet_HLH_glcm_Imc1	wavelet_HLL_ngtdm_Strength	wavelet_LHL_firstorder_Median
	DL_9	DL_11	DL_29						
**DLM**	DTL_0	DTL_1	DTL_2	DTL_4	DTL_11	DTL_27	DTL_31		
	DL_9	DL_11	DL_29						
**CSM**	original_firstorder_10Percentile_h3	original_firstorder_Median_h3	original_ngtdm_Strength_h3	original_shape_Flatness_h4	original_shape_LeastAxisLength_h4				
	3D maximum diameter	Density	Shape						
**RDLM**	log_sigma_1_0_mm_3D_glcm_Imc2	log_sigma_1_0_mm_3D_glcm_InverseVariance	log_sigma_1_0_mm_3D_gldm_DependenceVariance	original_shape_Sphericity	wavelet_HHH_firstorder_Skewness	wavelet_HHL_firstorder_Skewness	wavelet_HLH_glcm_Imc1	wavelet_HLL_ngtdm_Strength	wavelet_LHL_firstorder_Median

	DTL_0	DTL_1	DTL_2	DTL_4	DTL_11	DTL_27	DTL_31		
	DL_9	DL_11	DL_29						
**RCSM**	log_sigma_1_0_mm_3D_glcm_Imc2	log_sigma_1_0_mm_3D_glcm_InverseVariance	log_sigma_1_0_mm_3D_gldm_DependenceVariance	original_shape_Sphericity	wavelet_HHH_firstorder_Skewness	wavelet_HHL_firstorder_Skewness	wavelet_HLH_glcm_Imc1	wavelet_HLL_ngtdm_Strength	wavelet_LHL_firstorder_Median
	original_firstorder_10Percentile_h3	original_firstorder_Median_h3	original_ngtdm_Strength_h3	original_shape_Flatness_h4	original_shape_LeastAxisLength_h4				
	3D maximum diameter	Density	Shape						
**2DLCSM**	DTL_0	DTL_1	DTL_2	DTL_4	DTL_11	DTL_27	DTL_31		
	original_firstorder_10Percentile_h3	original_firstorder_Median_h3	original_ngtdm_Strength_h3	original_shape_Flatness_h4	original_shape_LeastAxisLength_h4				
	3D maximum diameter	Density	Shape						
**3DLCSM**	DL_9	DL_11	DL_29						
	original_firstorder_10Percentile_h3	original_firstorder_Median_h3	original_ngtdm_Strength_h3	original_shape_Flatness_h4	original_shape_LeastAxisLength_h4				
	3D maximum diameter	Density	Shape						
**RDLCSM**	log_sigma_1_0_mm_3D_glcm_Imc2	log_sigma_1_0_mm_3D_glcm_InverseVariance	log_sigma_1_0_mm_3D_gldm_DependenceVariance	original_shape_Sphericity	wavelet_HHH_firstorder_Skewness	wavelet_HHL_firstorder_Skewness	wavelet_HLH_glcm_Imc1	wavelet_HLL_ngtdm_Strength	wavelet_LHL_firstorder_Median
	DTL_0	DTL_1	DTL_2	DTL_4	DTL_11	DTL_27	DTL_31		
	DL_9	DL_11	DL_29						
	original_firstorder_10Percentile_h3	original_firstorder_Median_h3	original_ngtdm_Strength_h3	original_shape_Flatness_h4	original_shape_LeastAxisLength_h4				
	3D maximum diameter	Density	Shape						

Log indicates Laplacian of Gaussian

GLCM,Gray level co-occurrence matrix;NGTDM,Neighboring Gray Tone Difference Matrix;
For wavelet filtration,"H" and "L" represent high pass filter and low pass filter on the x,y,z directions.
The feature name suffix _n in the subregion represents the subregion sequence number
R2DLM (radiomics + 2D deep learning); R3DLM (radiomics + 3D deep learning); DLM (2D deep learning + 3D deep learning); CSM (clinical-visual radiological + tumor subregion)
RDLM (radiomics + 2D deep learning + 3D deep learning); RCSM (radiomics + clinical-visual radiological + tumor subregion); 2DLCSM (2D deep learning + clinical-visual radiological + tumor subregion); 3DLCSM (3D deep learning + clinical-visual radiological + tumor subregion)
RDLCSM (radiomics + 2D deep learning + 3D deep learning + clinical-visual radiological + tumor subregion)

Supplementary Table 5:The performance comparison of different models in the prospective cohort

Model	AUC (95%CI)	ACC	SEN	SPE	PPV	NPV
RDLM	0.825 (0.588-1.000)	0.833	0.857	0.818	0.750	0.900
2DLCSM	0.792 (0.542-1.000)	0.722	0.857	0.636	0.600	0.875
RDLCSM	0.909 (0.771-1.000)	0.889	1.000	0.818	0.778	1.000

RDLM (radiomics + 2D deep learning + 3D deep learning); 2DLCSM (2D deep learning + clinical-visual radiological + tumor subregion)
RDLCSM (radiomics + 2D deep learning + 3D deep learning + clinical-visual radiological + tumor subregion)
AUC, area under the summary receiver operating characteristic curve; CI, confidence interval.
ACC, accuracy; SEN, sensitivity; SPE, specificity.
PPV, positive predictive value; NPV,negative predictive value.

Supplementary Table 6: Summary of radiologists diagnostic performance

		AUC (95%CI)	ACC	SEN	SPE	PPV	NPV
**Junior**							
	Without model	0.640 (0.613-0.707)	0.639	0.643	0.636	0.551	0.720
	With RDLM	0.792 (0.735-0.849)	0.795	0.774	0.810	0.739	0.838
	With 2DLCSM	0.752 (0.691-0.812)	0.756	0.726	0.777	0.693	0.803
	With RDLCSM	0.832 (0.714-0.902)	0.834	0.821	0.843	0.784	0.872
**Senior**							
	Without model	0.723 (0.660-0.786)	0.727	0.702	0.744	0.656	0.783
	With RDLM	0.845 (0.794-0.896)	0.849	0.821	0.868	0.812	0.875
	With 2DLCSM	0.776 (0.718-0.835)	0.785	0.726	0.826	0.744	0.813
	With RDLCSM	0.861 (0.811-0.945)	0.863	0.845	0.876	0.826	0.891

RDLM (radiomics + 2D deep learning + 3D deep learning); 2DLCSM (2D deep learning + clinical-visual radiological + tumor subregion)
RDLCSM (radiomics + 2D deep learning + 3D deep learning + clinical-visual radiological + tumor subregion)
AUC, area under the summary receiver operating characteristic curve; CI, confidence interval.
ACC, accuracy; SEN, sensitivity; SPE, specificity.
PPV, positive predictive value; NPV,negative predictive value.

Supplementary Figure 1:Definition of visual radiological features

Lobulated

Absent

Present

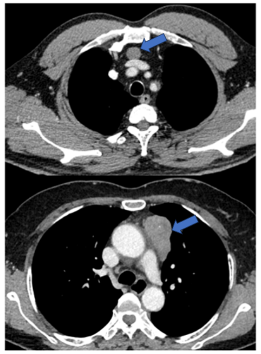

Primary site

Middle

Side

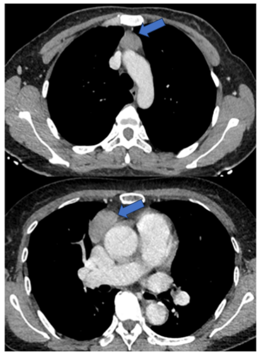

Density uniformity

uniform

Nonuniform

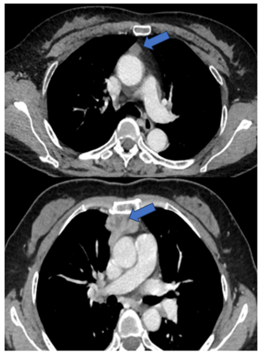

Shape

Regular

Irregular

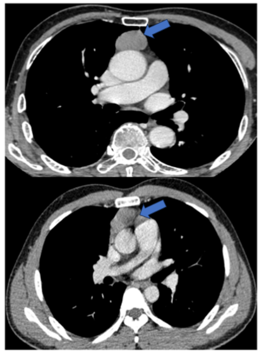

Calcification

Absent

Present

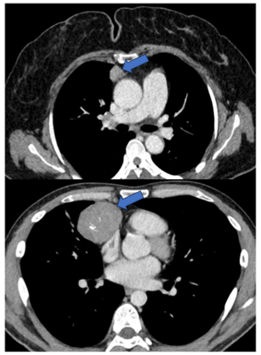

Boundary clarity

Clear

Vague

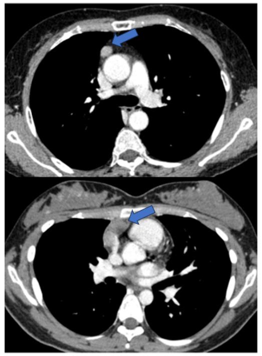

2D maximum
axis diameter

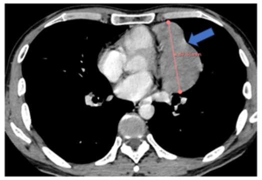

3D maximum
axis diameter

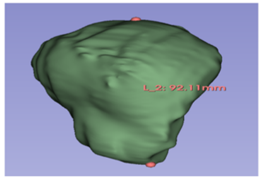

The blue arrow indicates lesion.

Supplementary Figure 2:Automatic segmentation process and result. (A). Automatic segmentation model training process index change curve. (B). Examples of segmentation in the test set.

A

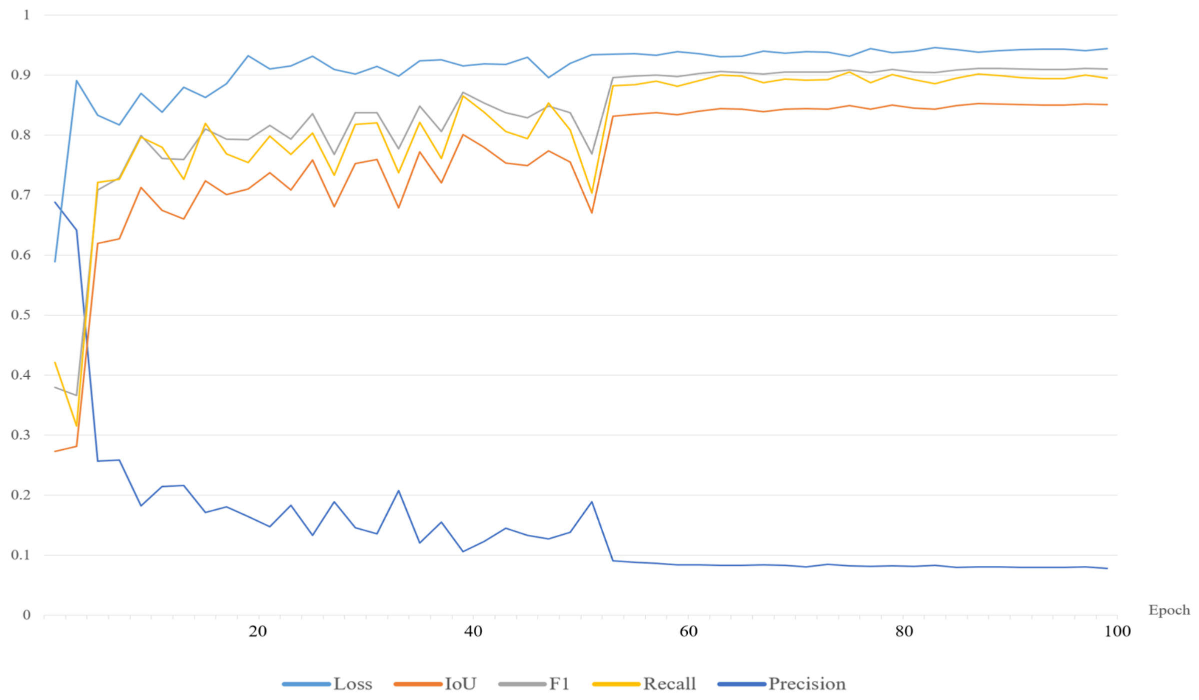

B

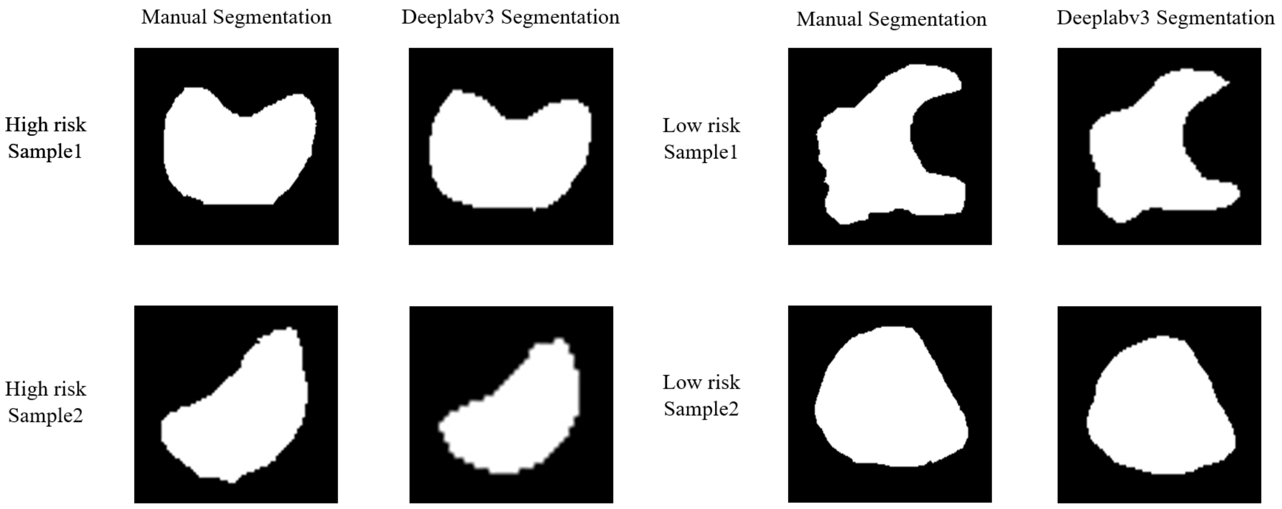

Supplementary Figure 3: LASSO radiomics features selection. (A). LASSO coefficient path: The figure shows the variation of characteristic coefficients corresponding to different values of regularization parameter (λ) in the LASSO algorithm. The abscissa represents the value of the regularization parameter, and the ordinate represents the absolute value or the scaled value of the characteristic coefficients. It can be known which features' coefficients tend to be sparse (become zero) with the increase of regularization parameters, so as to achieve the purpose of feature screening. (B). LASSO regularization path: This figure shows the model fitting effect corresponding to different values of the λ in the LASSO algorithm. The abscissa represents the values of the regularization parameters, and the ordinate represents the performance metrics of the model. By looking at this plot, one can find a suitable regularization parameter value that allows the model to have fewer features while maintaining its predictive performance. (C). Feature coefficient ranking: the coefficients of each feature were selected by LASSO to rank the importance

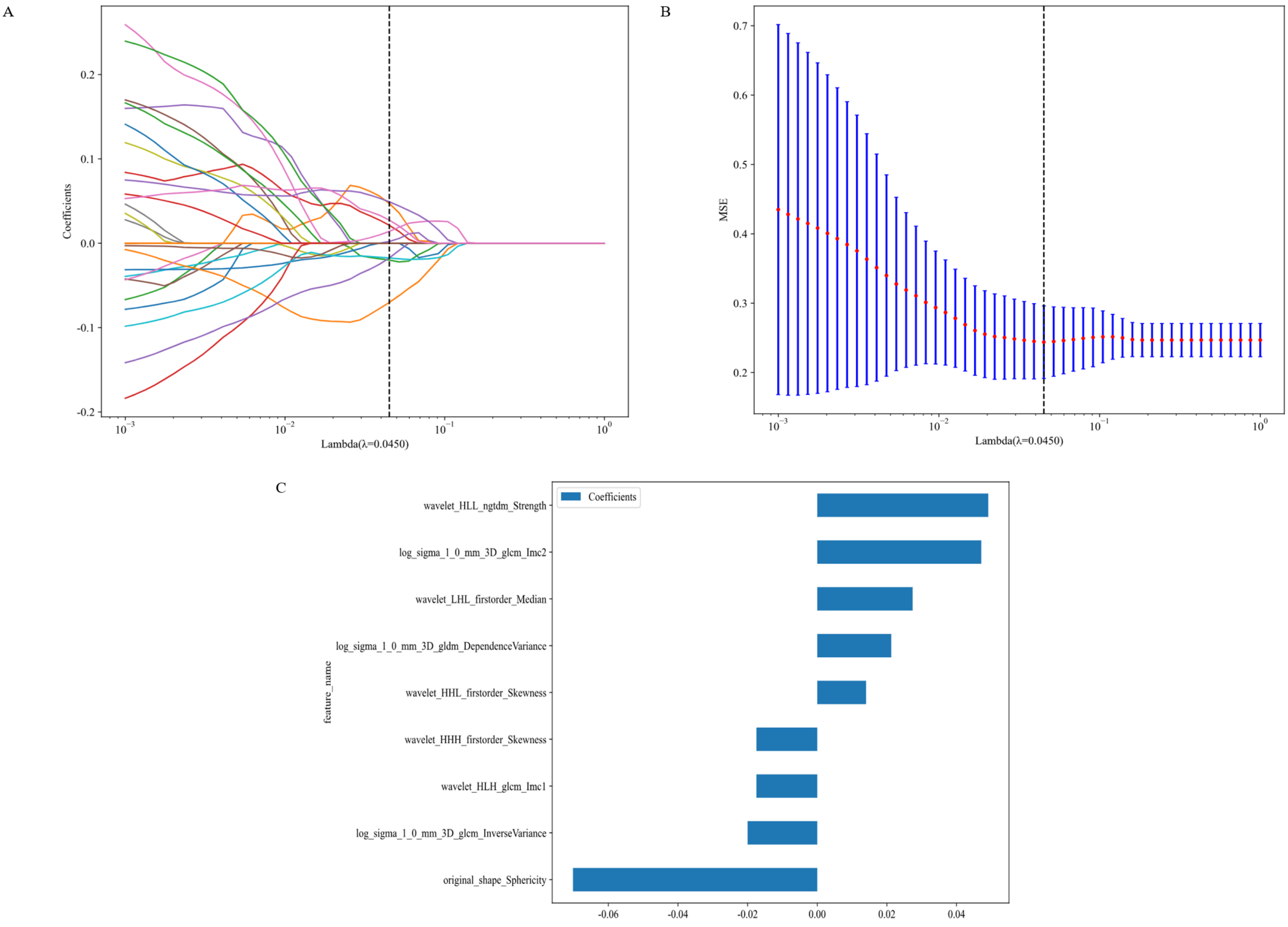

Supplementary Figure 4: LASSO subregion features selection. (A). LASSO coefficient path: The figure shows the variation of characteristic coefficients corresponding to different values of regularization parameter (λ) in the LASSO algorithm. The abscissa represents the value of the regularization parameter, and the ordinate represents the absolute value or the scaled value of the characteristic coefficients. It can be known which features' coefficients tend to be sparse (become zero) with the increase of regularization parameters, so as to achieve the purpose of feature screening. (B). LASSO regularization path: This figure shows the model fitting effect corresponding to different values of the λ in the LASSO algorithm. The abscissa represents the values of the regularization parameters, and the ordinate represents the performance metrics of the model. By looking at this plot, one can find a suitable regularization parameter value that allows the model to have fewer features while maintaining its predictive performance. (C). Feature coefficient ranking: the coefficients of each feature were selected by LASSO to rank the importance

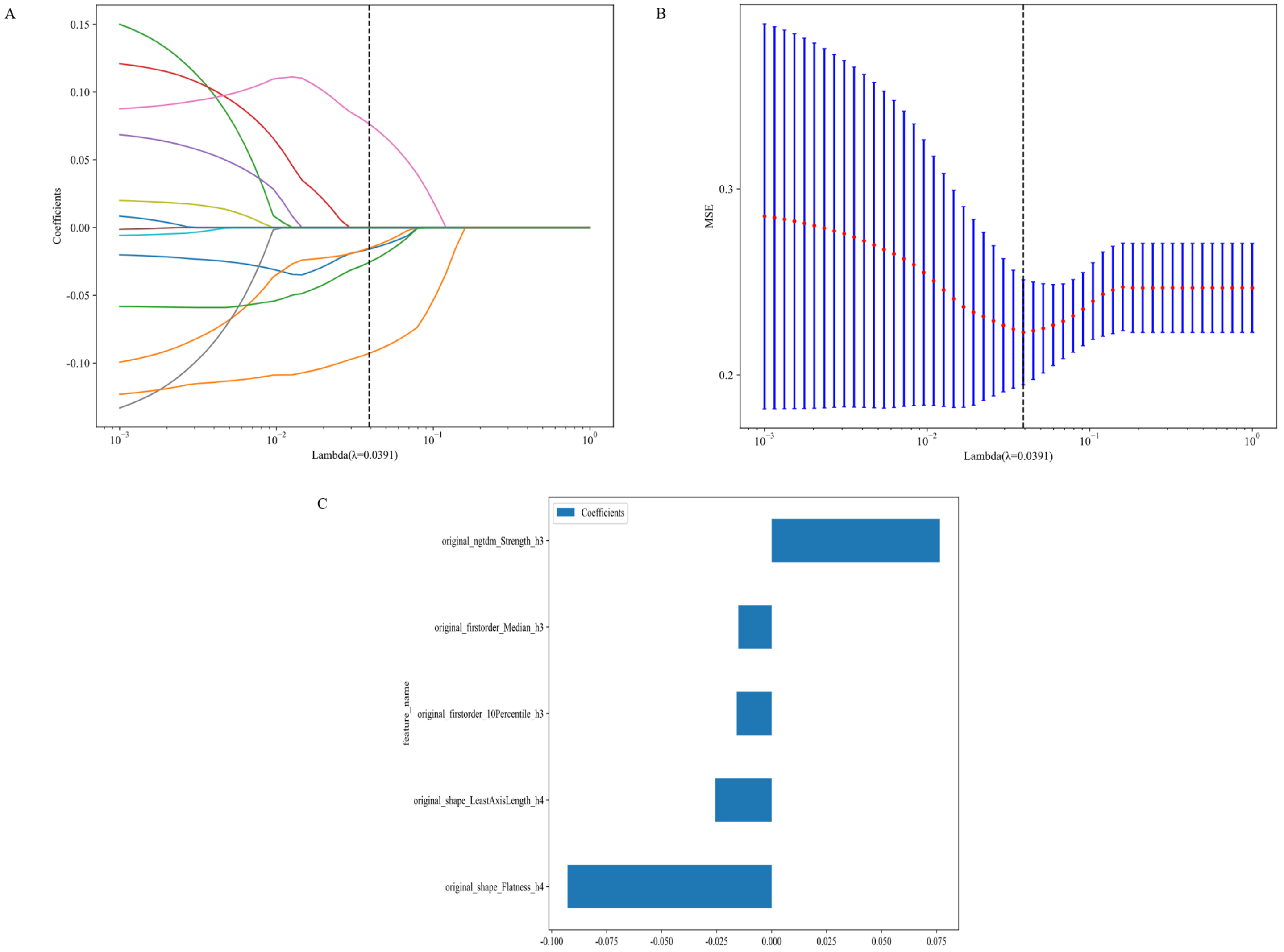

Supplementary Figure 5: DL features selection. Figures A, B, and C show the 2D deep learning model using LASSO for feature selection. Figures D, E, and F show the 3D deep learning model using LASSO for feature selection. DTL and DL are used for prefix naming of 2D and 3D DL features, respectively. For example, DTL_27 is the 28th 2D deep learning feature after PCA (the first one is named 0), and similarly, DL_29 represents the 30th 3D deep learning feature after PCA.

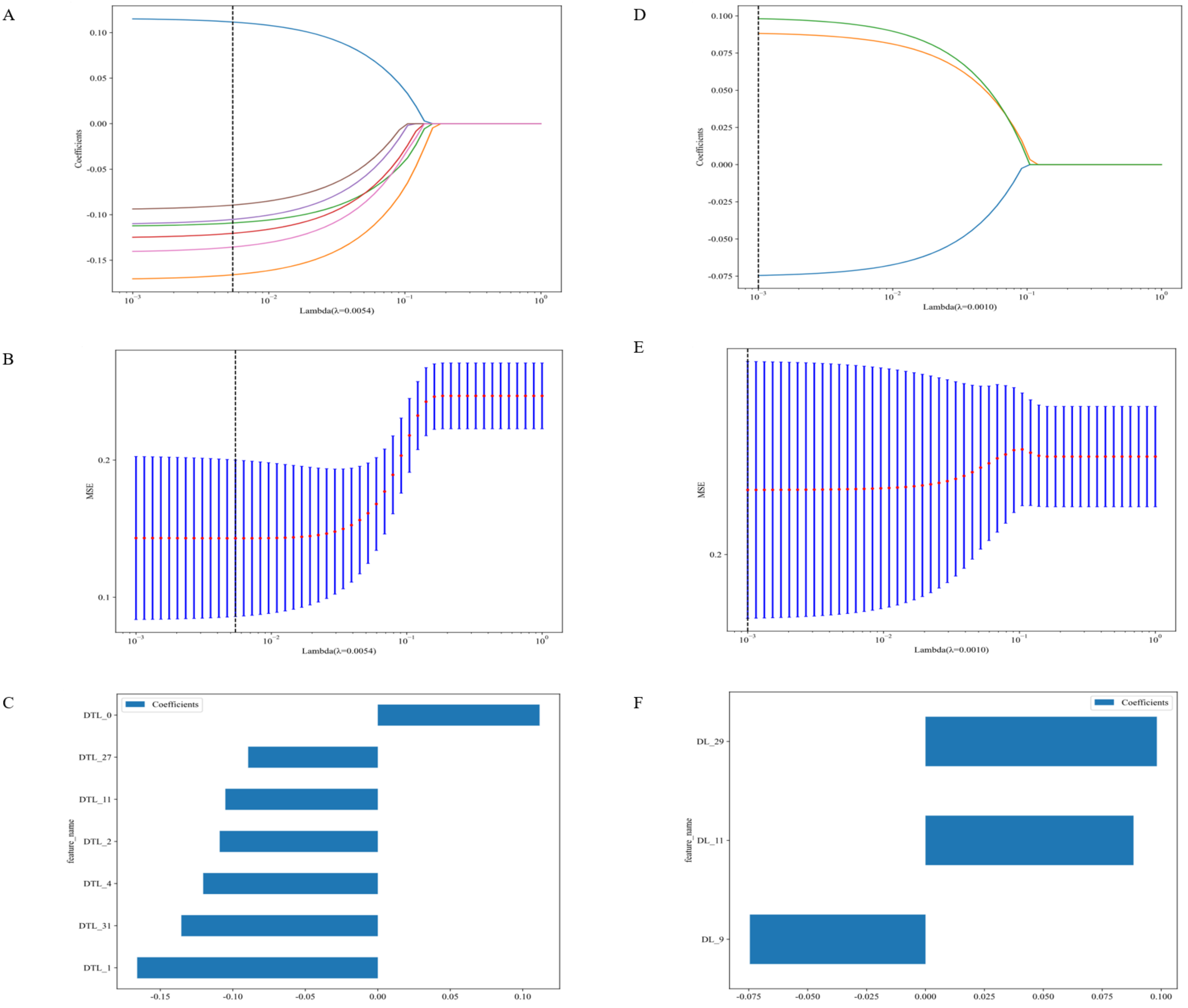

Supplementary Figure 6: ROC curves of RM

The ROC curves of RM in the training cohort (A), internal validation cohort (B), external validation cohort1 (C), and external validation cohort2 (D).

AUC, area under the summary receiver operating characteristic curv; CI, confidence interval; KNN, K-Nearest Neighbor; LightGBM, Light Gradient Boosting Machine; MLP, Multilayer Perceptron.

Training cohort (n=100); Internal validation cohort (n=44); External validation cohort1 (n=44); External validation cohort2 (n=17).

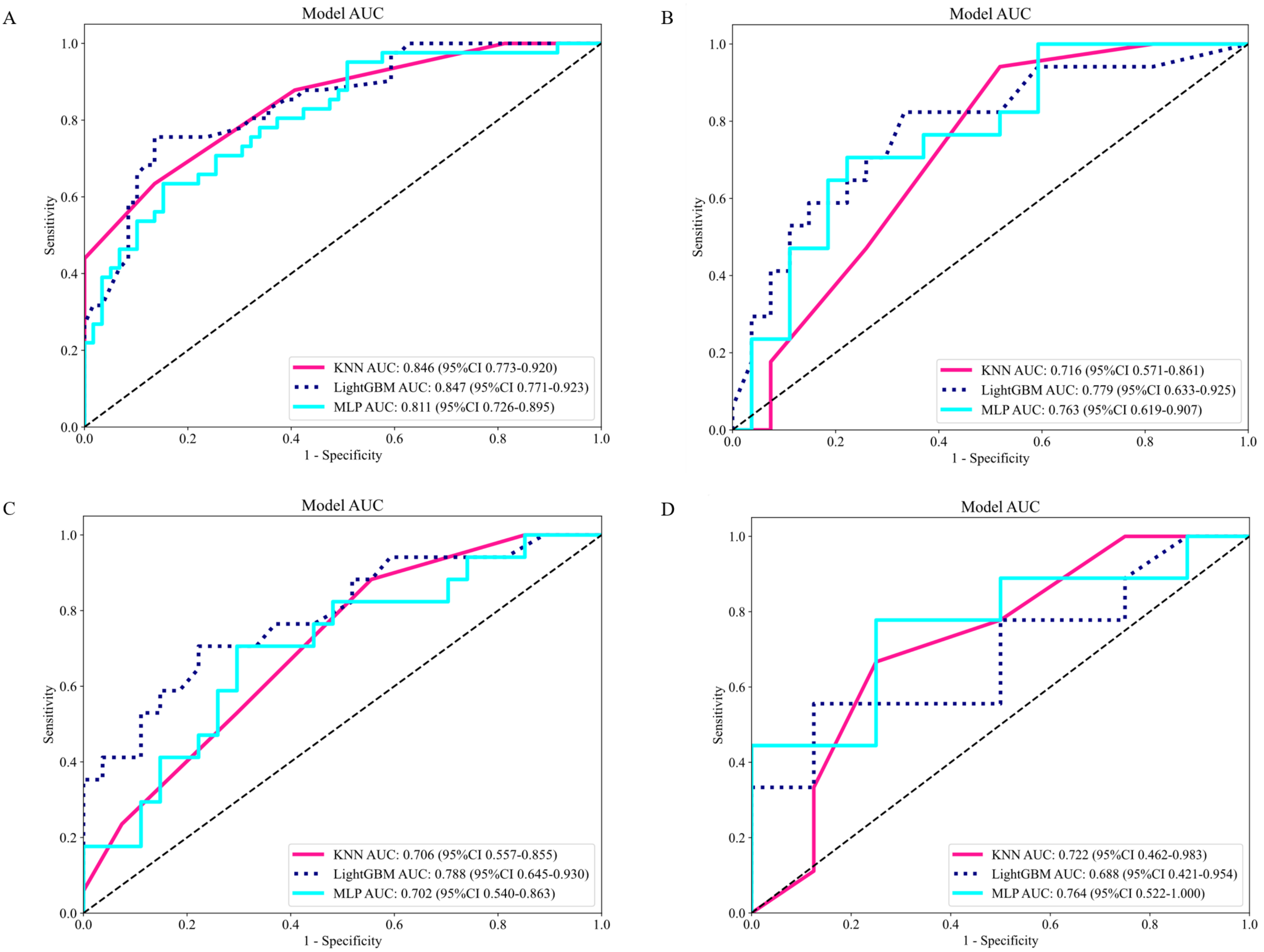

Supplementary Figure 7: ROC curves of 2DLM

The ROC curves of 2DLM in the training cohort (A), internal validation cohort (B), external validation cohort1 (C), and external validation cohort2 (D).

AUC, area under the summary receiver operating characteristic curv; CI, confidence interval. Training cohort (n=100); Internal validation cohort (n=44); External validation cohort1 (n=44); External validation cohort2 (n=17).

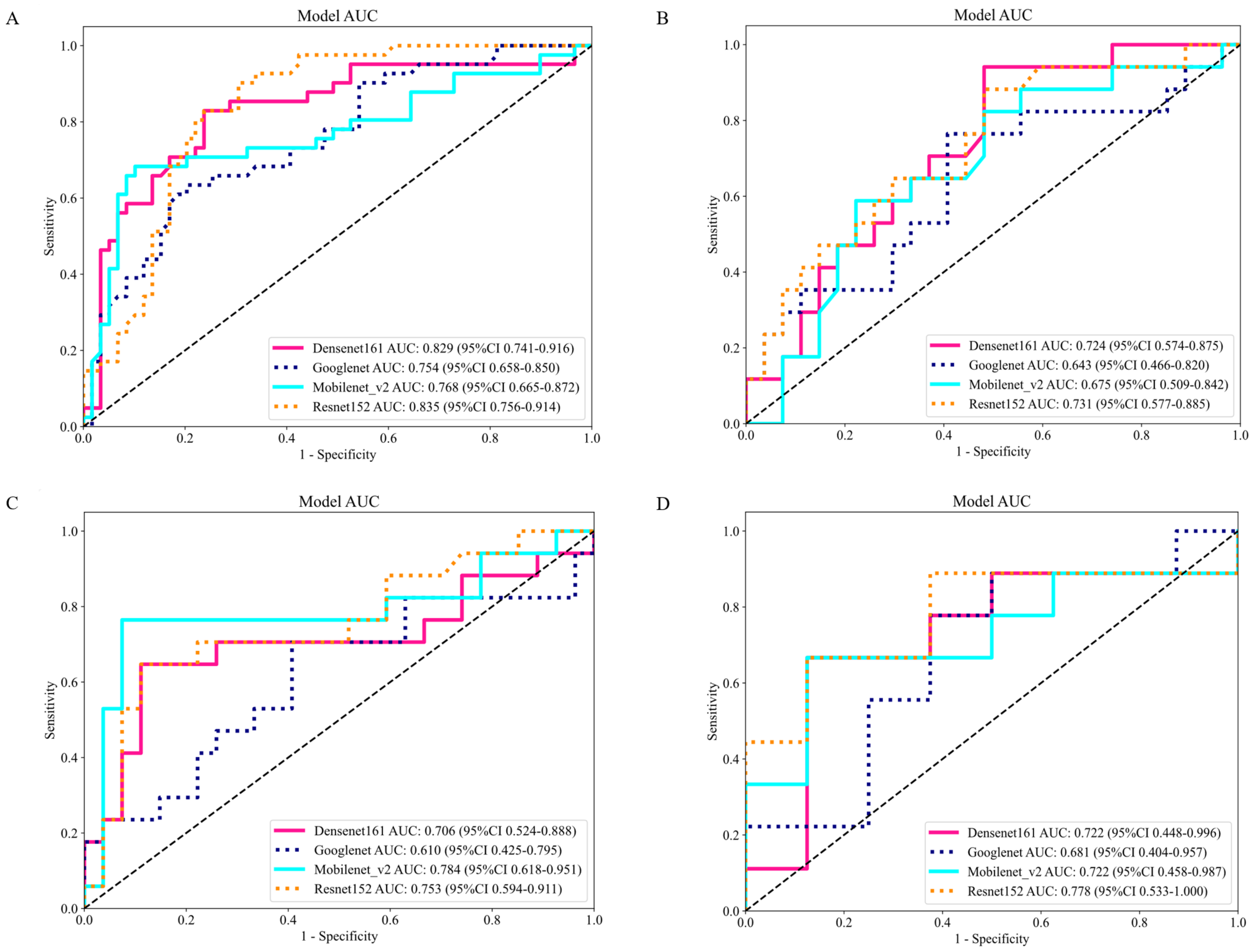

Supplementary Figure 8: ROC curves of 3DLM

The ROC curves of 3DLM in the training cohort (A), internal validation cohort (B), external validation cohort1 (C), and external validation cohort2 (D).

AUC, area under the summary receiver operating characteristic curv; CI, confidence interval; ViT, Vision Transformer.

Training cohort (n=100); Internal validation cohort (n=44); External validation cohort1 (n=44); External validation cohort2 (n=17).

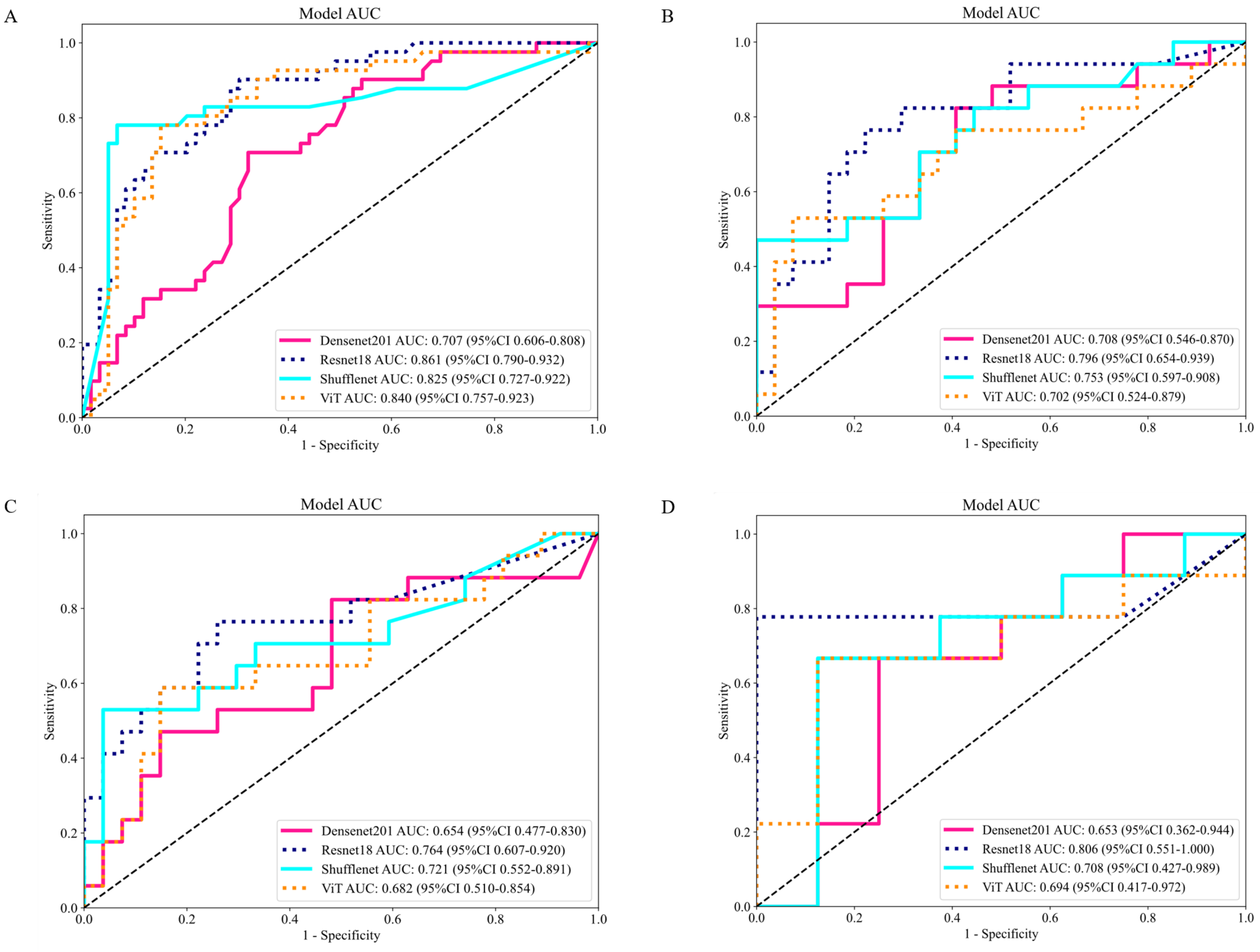

Supplementary Figure 9: ROC curves of two types of feature fusion models

The ROC curves of two types of feature fusion models in the training cohort (A), internal validation cohort (B), external validation cohort1 (C), and external validation cohort2 (D).

Training cohort (n=100); Internal validation cohort (n=44); External validation cohort1 (n=44); External validation cohort2 (n=17).

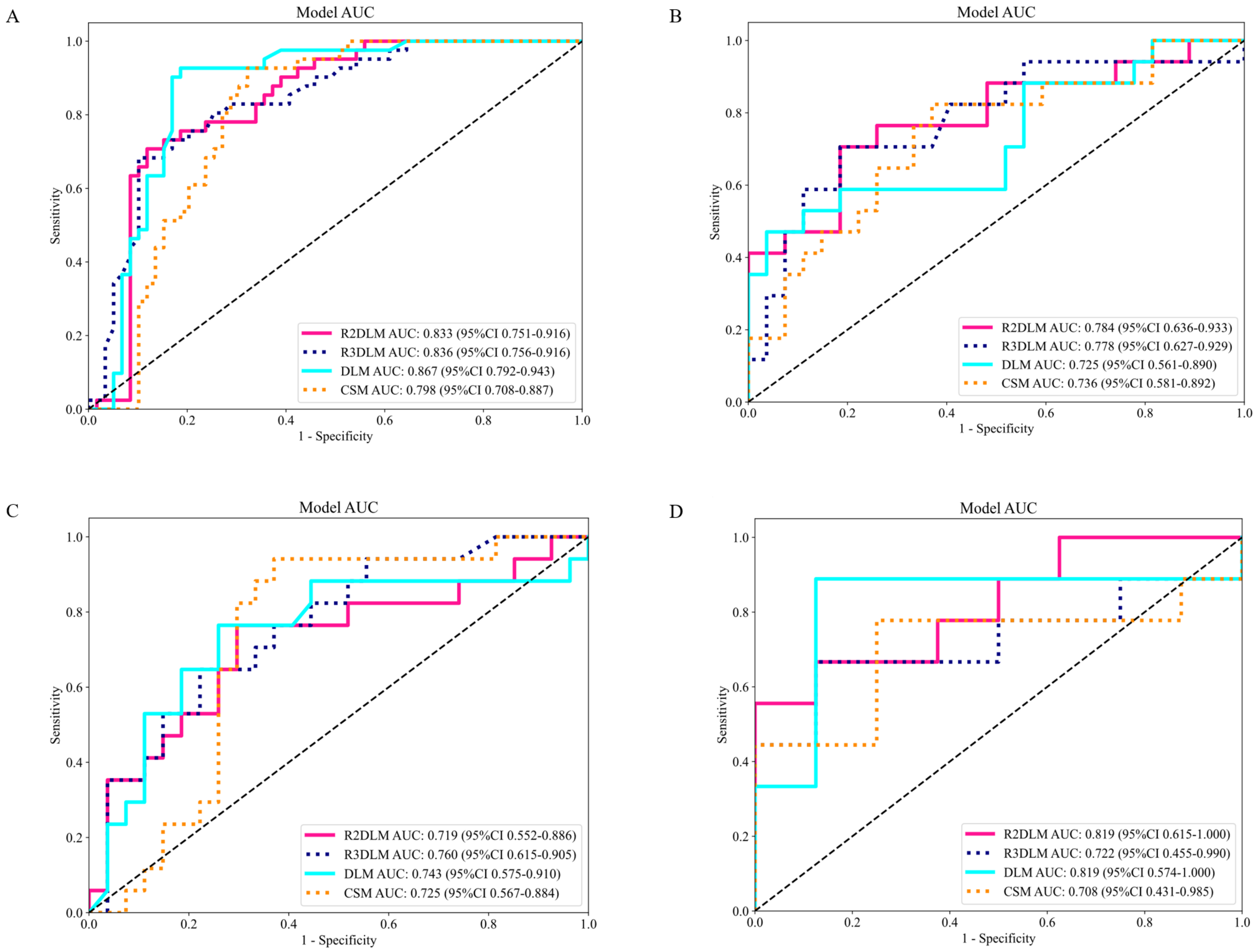

Supplementary Figure 10: Calibration curve

The calibration curves of different models in the training cohort (A), internal validation cohort (B), external validation cohort1 (C), and external validation cohort2 (D).

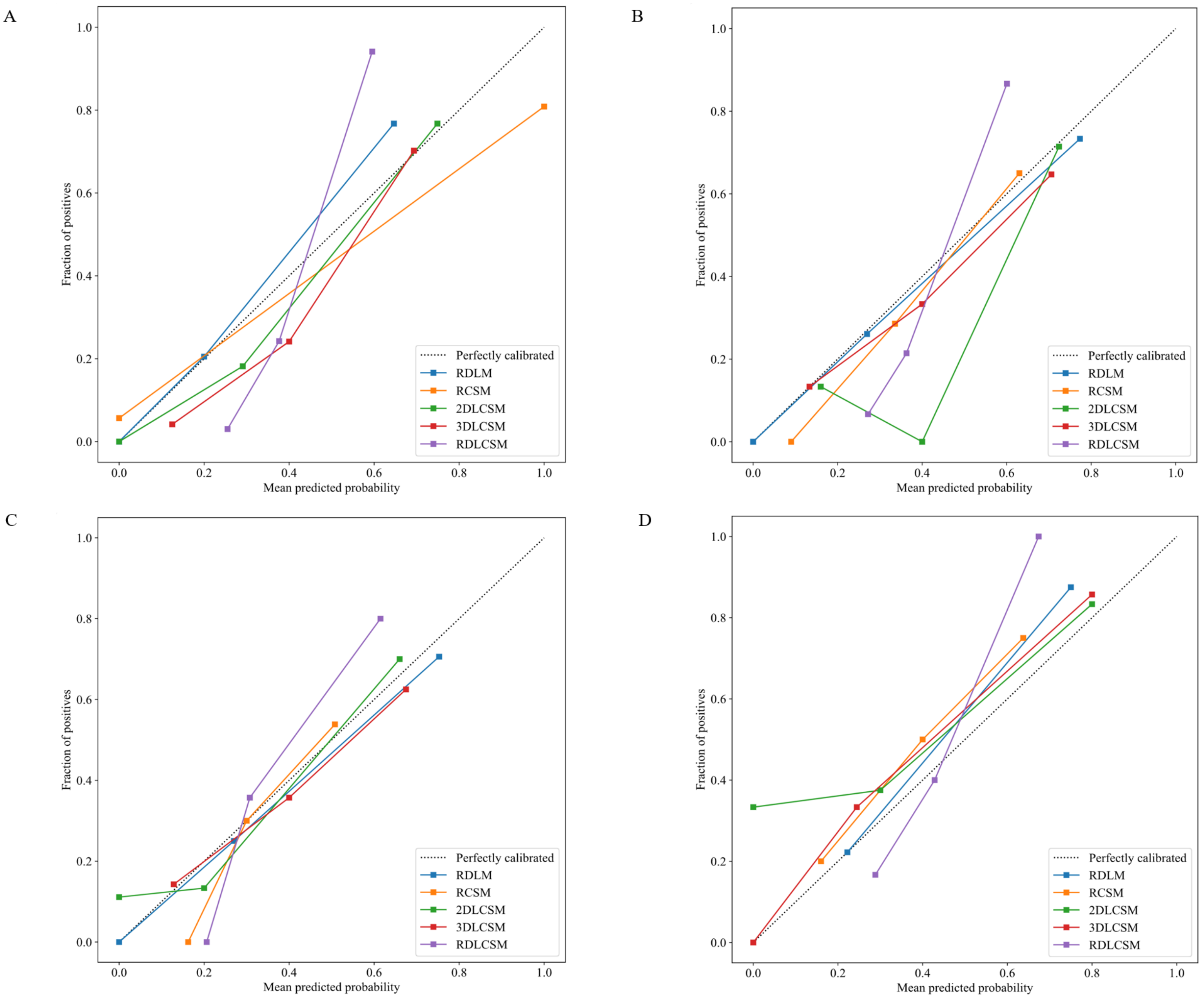

Supplementary Figure 11: Decision curve analysis

The decision curve analysis of different models in the training cohort (A), internal validation cohort (B), external validation cohort1 (C), and external validation cohort2 (D).

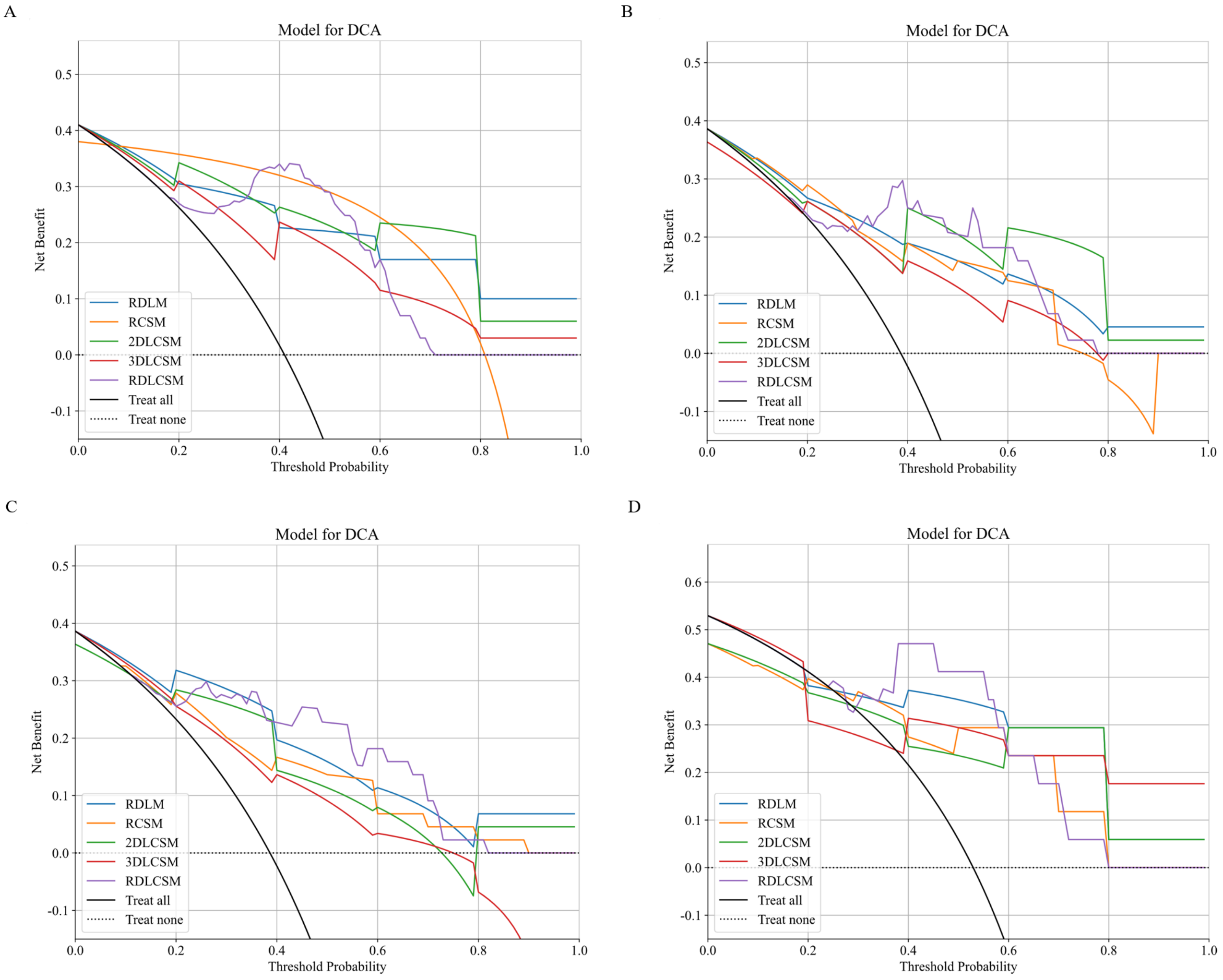

Supplementary Figure 12:Stratified analysis

The ROC curve of RDLCSM, 2DLCSM, RDLM in stratified analysis of different gender (A), age (B), and CT scan thickness (C).

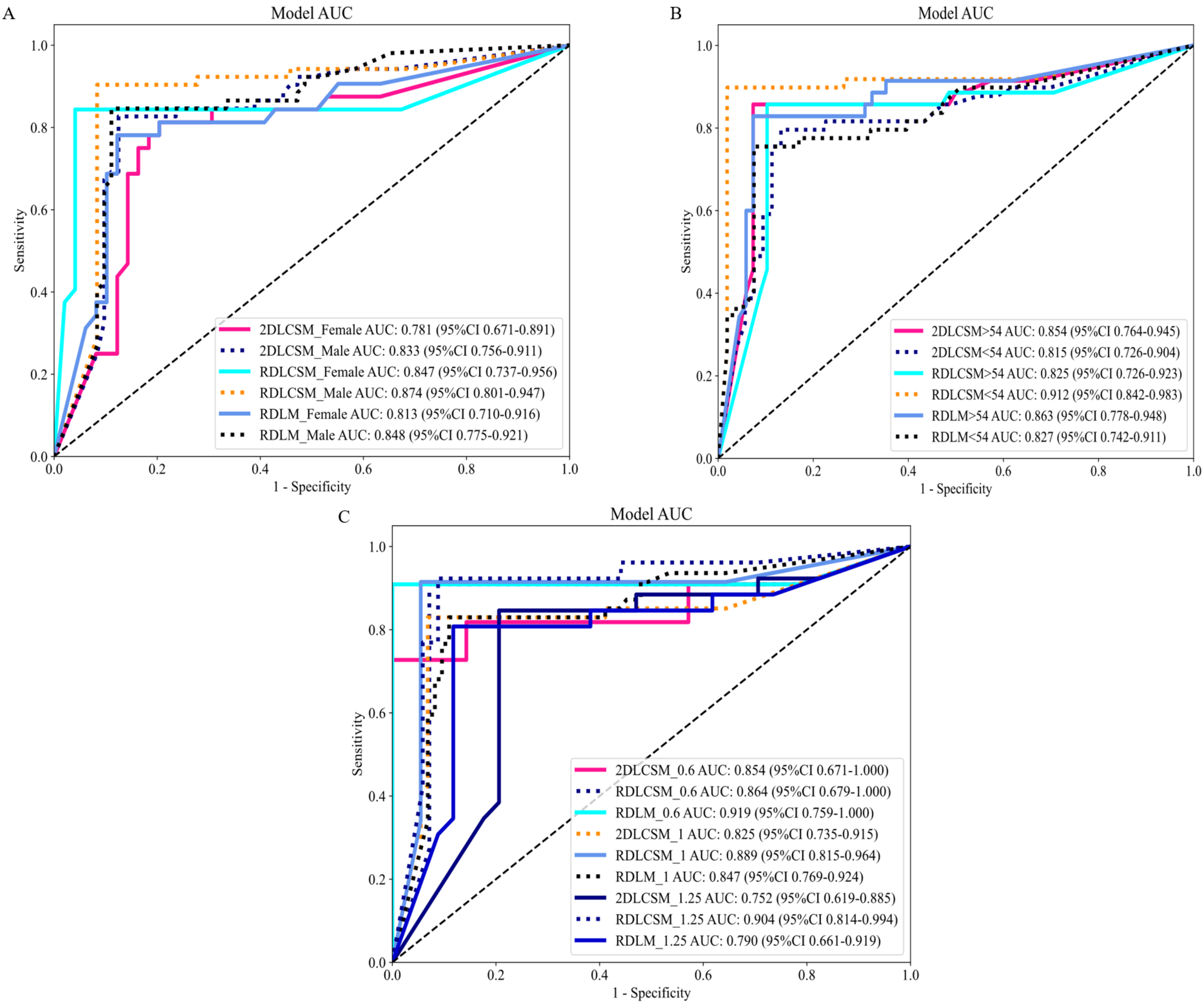

Supplementary Figure 13: With or without model-assisted diagnostic performance of radiologists

Diagnostic performance of high and low seniority radiologists with or without the assistance of different models (RDLM, 2DLCSM, RDLCSM).

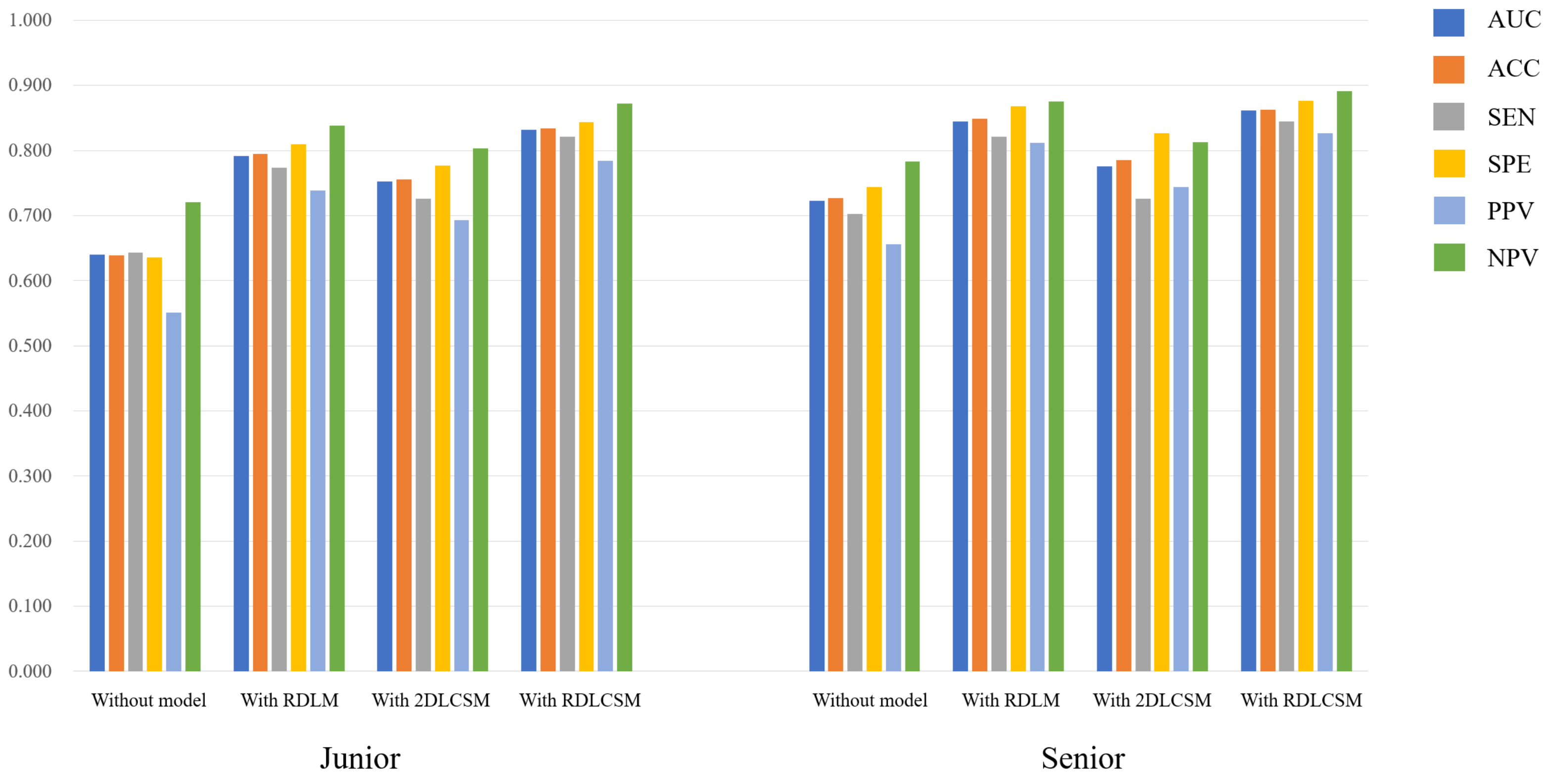

Supplement: Supplementary file 1 [file AO-64-42982-s1.pdf]
